# In vivo creation of plasmid pCRT01 and its use for the construction of carotenoid-producing *Paracoccus* spp. strains that grow efficiently on industrial wastes

**DOI:** 10.1186/s12934-020-01396-z

**Published:** 2020-07-13

**Authors:** Anna Maj, Lukasz Dziewit, Lukasz Drewniak, Maciej Garstka, Tomasz Krucon, Katarzyna Piatkowska, Katarzyna Gieczewska, Jakub Czarnecki, Ewa Furmanczyk, Robert Lasek, Jadwiga Baj, Dariusz Bartosik

**Affiliations:** 1 Department of Bacterial Genetics, Institute of Microbiology, Faculty of Biology, University of Warsaw, Miecznikowa 1, 02-096 Warsaw, Poland; 2 Department of Environmental Microbiology and Biotechnology, Institute of Microbiology, Faculty of Biology, University of Warsaw, Miecznikowa 1, 02-096 Warsaw, Poland; 3 Department of Metabolic Regulation, Institute of Biochemistry, Faculty of Biology, University of Warsaw, Miecznikowa 1, 02-096 Warsaw, Poland; 4 Department of Plant Anatomy and Cytology, Institute of Experimental Plant Biology and Biotechnology, Faculty of Biology, University of Warsaw, Miecznikowa 1, 02-096 Warsaw, Poland; 5 Bacterial Genome Plasticity, Department of Genomes and Genetics, Institut Pasteur, Paris, France; 6 Department of Plant Protection from Pests, Research Institute of Horticulture, Skierniewice, Poland

**Keywords:** Carotenoids, Astaxanthin, β-carotene, *Paracoccus marcusii*, *Paracoccus aminophilus*, *Paracoccus kondratievae*, Plasmid pCRT01

## Abstract

**Background:**

Carotenoids are natural tetraterpene pigments widely utilized in the food, pharmaceutical and cosmetic industries. Currently, chemical synthesis of these compounds outperforms their production in *Escherichia coli* or yeast due to the limited efficiency of the latter. The use of natural microbial carotenoid producers, such as bacteria of the genus *Paracoccus* (*Alphaproteobacteria*), may help to optimize this process. In order to couple the ability to synthesize these pigments with the metabolic versatility of this genus, we explored the possibility of introducing carotenoid synthesis genes into strains capable of efficient growth on simple low-cost media.

**Results:**

We constructed two carotenoid-producing strains of *Paracoccus* carrying a new plasmid, pCRT01, which contains the carotenoid synthesis gene locus *crt* from *Paracoccus marcusii* OS22. The plasmid was created in vivo via illegitimate recombination between *crt*-carrying vector pABW1 and a natural “paracoccal” plasmid pAMI2. Consequently, the obtained fusion replicon is stably maintained in the bacterial population without the need for antibiotic selection. The introduction of pCRT01 into fast-growing “colorless” strains of *Paracoccus aminophilus* and *Paracoccus kondratievae* converted them into efficient producers of a range of both carotenes and xanthophylls. The exact profile of the produced pigments was dependent on the strain genetic background. To reduce the cost of carotenoid production in this system, we tested the growth and pigment synthesis efficiency of the two strains on various simple media, including raw industrial effluent (coal-fired power plant flue gas desulfurization wastewater) supplemented with molasses, an industrial by-product rich in sucrose.

**Conclusions:**

We demonstrated a new approach for the construction of carotenoid-producing bacterial strains which relies on a single plasmid-mediated transfer of a pigment synthesis gene locus between *Paracoccus* strains. This strategy facilitates screening for producer strains in terms of synthesis efficiency, pigment profile and ability to grow on low-cost industrial waste-based media, which should increase the cost-effectiveness of microbial production of carotenoids.

## Background

In many cases, microbial production of commercially valuable compounds has advantages over their chemical synthesis. Many compounds used in industrial applications are complex molecules that naturally occur in living organisms. They are not easily reproducible by chemical processes, which makes their chemical synthesis expensive. In addition, the extraction of compounds from their natural sources is often not efficient enough to be profitable. Furthermore, the use of naturally-produced additives (e.g. in the pharmaceutical, cosmetic or food industries) is often considered healthier by consumers. For these reasons, genetically-modified microorganisms that produce large amounts of specific complex compounds and can grow on simple low-cost media represent an attractive alternative.

Carotenoids belong to a large group of organic compounds called isoprenoids. They are hydrophobic unsaturated hydrocarbons formed by the condensation of C_5_ units, isopentenyl pyrophosphate (IPP) and its isomer dimethylallyl pyrophosphate (DMAPP), most often to C_40_ chains. Carotenoids are common natural pigments with rich colors ranging from yellow through orange to red, and even purple or brown [1].

Although carotenoids play a significant role in the functioning of all organisms as antioxidants and UV protective agents, they are only synthesized by certain groups. These include plants, algae and photosynthetic bacteria, in which they are necessary for protection of the photosynthetic machinery [2]. They are also synthesized by some fungi and non-photosynthetic bacteria [3, 4]. Carotenoids are not produced by humans, so are an important component of our diet. Some of these compounds are precursors of vitamin A (carotenoids containing β-rings, e.g. β-carotene, γ-carotene or cryptoxanthin), which is involved in the maintenance of membrane integrity, bone development, keeping the skin, hair and nails in a healthy condition, and importantly in vision–lutein and zeaxanthin are used as pigments of the macula located in the center of the human retina [5].

The use of carotenoids as dietary supplements or active compounds in pharmaceuticals has been shown to delay the onset and significantly reduce the risk of diseases such as atherosclerosis, cataracts, macular degeneration, multiple sclerosis and various types of cancer [6]. Astaxanthin was shown to hinder stomach colonization by *Helicobacter pylori* in mice, and to support and modulate the immune system [7].

Carotenoids are used in large quantities in food and cosmetics production. According to a recent report, the value of the global market of carotenoids in 2017 was 1.5 billion USD, and this is expected to reach 2.0 billion USD by 2022 [8], with β-carotene and astaxanthin as the two most profitable compounds [9].

Currently, the commercial production of carotenoids is primarily by multi-step chemical synthesis (around 90% of β-carotene and astaxanthin), with only a small quantity extracted from natural sources [9]. Microbiological methods of carotenoid production are a promising alternative to the processes used currently [9]. The optimization of methods based on natural (wild-type) unicellular carotenoid producers is unlikely to greatly increase pigment productivity, so alternative strategies using genetically modified organisms are being developed.

Most attempts to obtain microorganisms overproducing carotenoids have been based on the application of two model organisms: *Escherichia coli* and *Saccharomyces cerevisiae* [10]. The metabolic networks of this bacterium and yeast were augmented with heterologous pathways for the synthesis of both carotenoid precursors and the final pigments [11, 12]. When combined with adjustments of the biological, chemical and physical conditions (including gene expression and culture conditions) this strategy gave good results for β-carotene and lycopene production (in *E. coli* and yeast, respectively) [10]. However, despite significant progress in the optimization of carotenoid synthesis in these two microbial hosts, their metabolism was found to be unsuited to this task and overproduction caused toxic effects. There have been few studies on other microorganisms that could constitute better “platforms” for the production of these compounds via heterologous biosynthetic pathways [13].

Good candidates for this purpose are bacteria of the genus *Paracoccus* [14], among which numerous carotenoid producers have been identified, e.g. *Paracoccus marcusii* [15], *Paracoccus carotinifaciens* [16] and *Paracoccus haeundaensis* [17]. However, some pigment producing *Paracoccus* strains are not amenable to genetic manipulation and grow poorly in culture. In comparison, some “colorless” *Paracoccus* spp. grow robustly even on simple media, some of which contain methanol (a by-product of many industrial processes) as a carbon source. The use of these strains modified to carry carotenoid synthesis genes originating from closely related pigment producers may be a good strategy to obtain microorganisms for the efficient low-cost industrial production of natural carotenoids.

In this study, we used two “colorless”, methylotrophic and fast-growing *Paracoccus* strains, *P. aminophilus* JCM 7686 [18, 19] and *P. kondratievae* NCIBM 13773 [20], to express *P. marcusii* OS22 genes for carotenoid synthesis, which resulted in the efficient production of these compounds. In addition, a low-cost medium was developed to grow these strains, which can be utilized for biotechnological applications.

## Results

### Cloning of the *P. marcusii* OS22 *crt* gene cluster

Bacterial strain OS22 was isolated from the ancient Zloty Stok gold mine in Lower Silesia (Poland) in a study of microbial transformation of arsenic compounds [21]. This bacterium produces pigmented colonies with an intense orange color. Based on the results of comparative 16S rDNA sequence analyses the OS22 strain was classified as the species *P. marcusii* (*Alphaproteobacteria*), whose type strain DSM 11574 has the ability to produce astaxanthin–a carotenoid of commercial value. Our preliminary analysis using ultra-performance liquid chromatography (UPLC) confirmed that OS22 shares this property (see Additional file 1: Fig. S1 and Additional file 2: Table S1). However, further studies on carotenoid synthesis by this strain proved problematic. It grew poorly under laboratory conditions and the cells frequently lysed in liquid culture. Moreover, individually tested clones showed spontaneous loss of different endogenous plasmids, which revealed a strain prone to genomic instability. Therefore, an attempt was made to clone the OS22 genes responsible for astaxanthin synthesis (*crt*) as a CRT cassette in order to test their functionality in other hosts.

Since the genomic sequence of OS22 was unavailable, the *crt* genes were isolated from the OS22 genome using a multi-step approach. Based on the nucleotide sequence of the *crt locus* of the *P. marcusii* type strain (DSM 11574) (GenBank: Y15112), we designed a primer pair specific to *crtW*–the first gene in the *locus*. These primers enabled amplification of the complete *crtW* gene of OS22 using genomic DNA of this strain as the template. This DNA fragment was cloned into the *E.* *coli*-specific vector pABW1 (Km^r^), which is unable to replicate in *Paracoccus* spp. The resulting plasmid pABW1-crtW was then introduced into strain OS22 by conjugation. The presence of the *crtW* gene enabled sequence-targeted integration of this suicide plasmid into the host’s genome by means of homologous recombination. The entire OS22 CRT cassette was then isolated in a three-step process: (i) extraction of genomic DNA from the cointegrate-containing strain, (ii) digestion of isolated genomic DNA with restriction endonuclease EcoRI (no recognition sites within pABW1-crtW) to generate a restriction fragment containing the integrated plasmid and adjacent *crt* genes, and (iii) circularization of this DNA fragment by ligation in vitro to produce recombinant plasmid molecules that replicate in *E. coli* cells (Additional file 1: Fig. S2). Plasmid DNA was then isolated and analyzed by DNA sequencing to characterize the cloned genes.

### Characterization of the OS22 *crt* locus

**Fig. 1:**
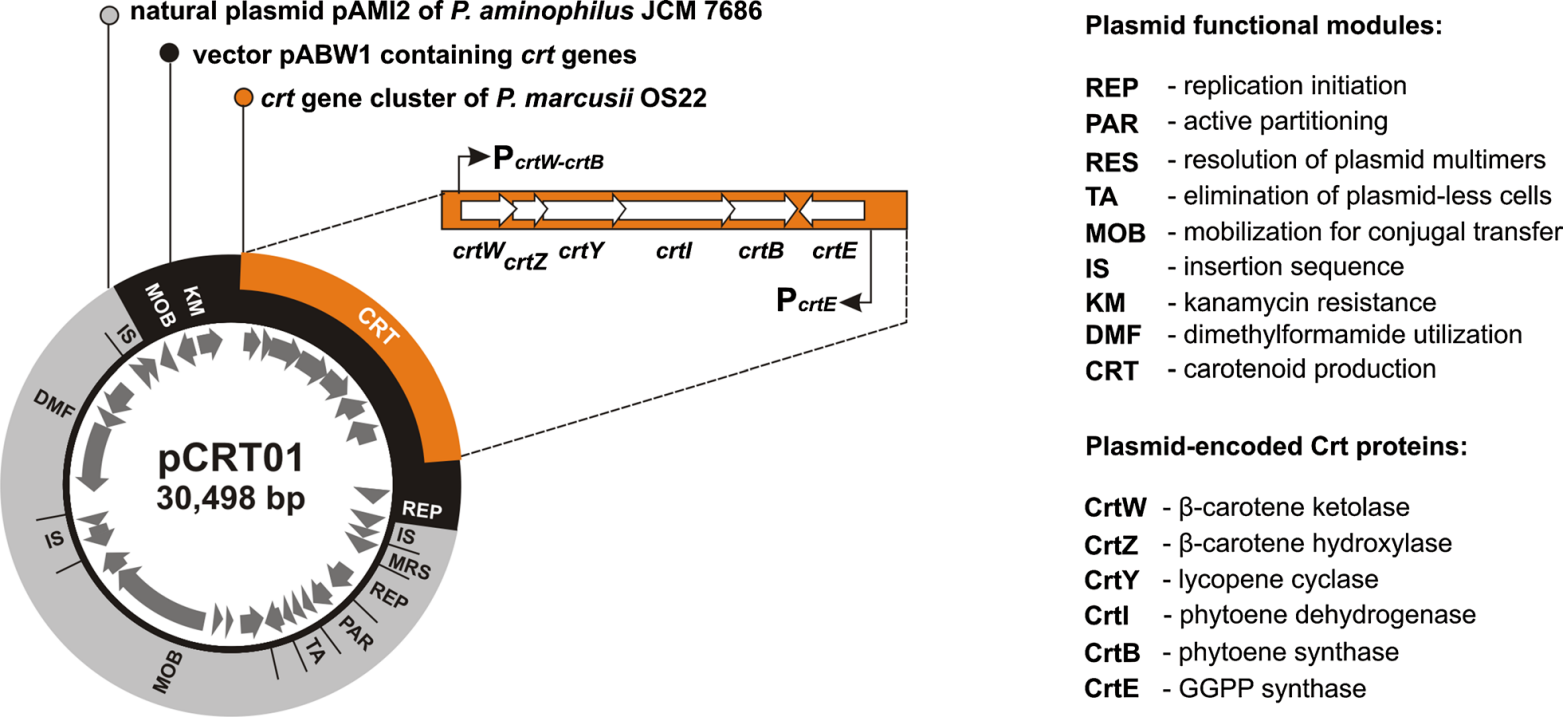
Plasmid pCRT01 and its *crt* gene cluster of *P. marcusii* OS22

**Fig. 2:**
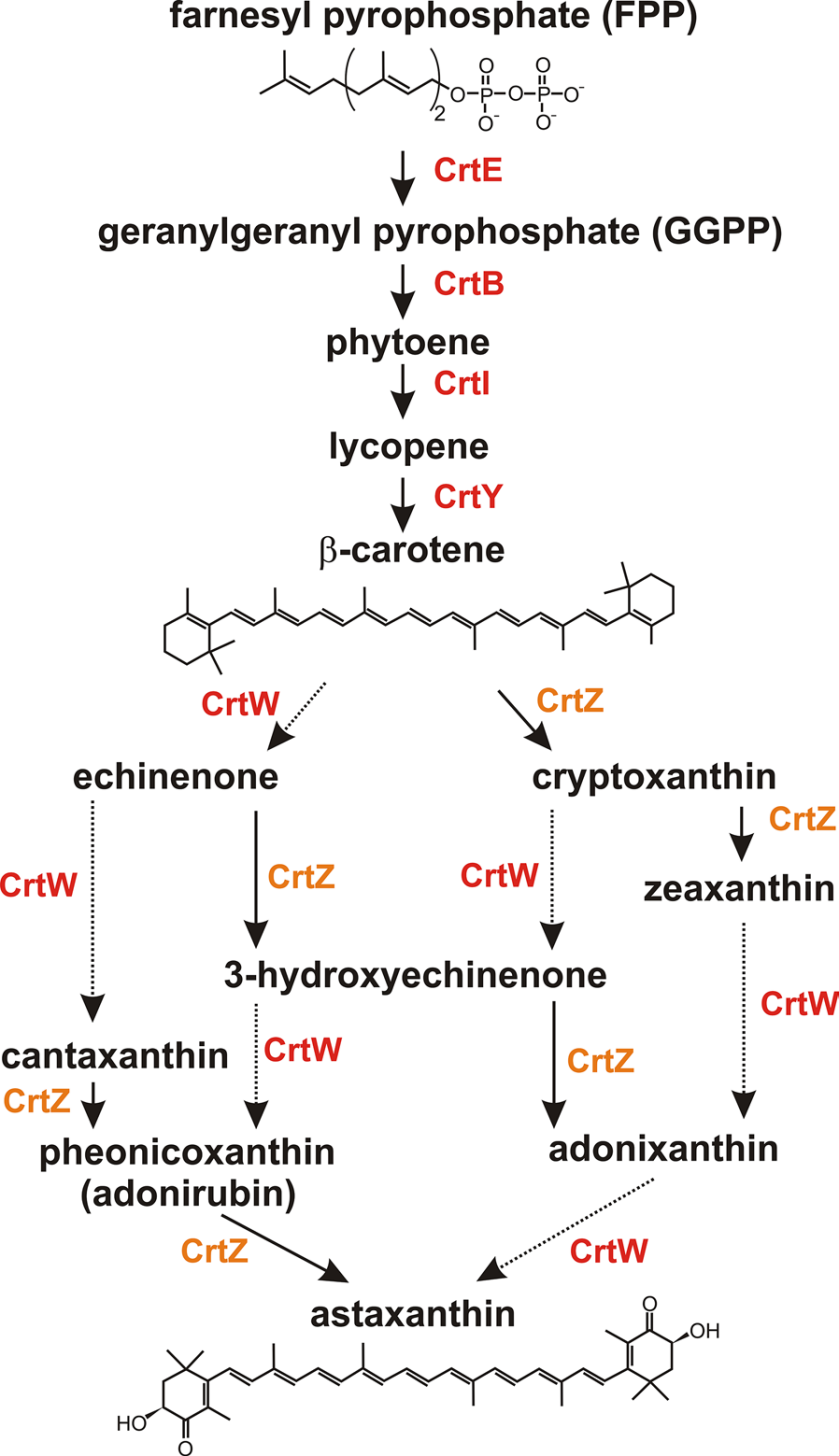
Bacterial carotenoid pigment biosynthesis pathway showing which Crt enzymes catalyze the different steps

Colonies of *E. coli* DH5α containing the obtained plasmid (pABW1-crt) changed their color to light orange after prolonged incubation. This observation suggested that the introduced plasmid could initiate pigment production in this host, although at very low efficiency. DNA sequencing revealed that the cloned OS22 DNA region contained a cluster of *crt* genes. This region displayed very high nucleotide sequence identity (96–98%) to the *crt locus* of *P. marcusii* DSM 11574, the carotenoid biosynthesis gene clusters of *Paracoccus* sp. N81106 [22] and *P. haeundaensis* [17], the chromosome of *Paracoccus* sp. Arc7-R13 (GenBank: CP034810) as well as the genomes of *Paracoccus* sp. 228 [23] and *Paracoccus* sp. S4493 [24]. All of these *crt loci* contain 6 common genes in synteny, *crtWZYIBE* (Fig. 1), encoding predicted polypeptides homologous to Crt proteins that have defined enzymatic activities, which play key roles in subsequent steps of carotenoid synthesis (Fig. 2). These are β-carotene oxygenase (CrtW), β-carotene hydroxylase (CrtZ), lycopene cyclase (CrtY), phytoene desaturase (CrtI), phytoene synthase (CrtB) and geranylgeranyl diphosphate (GGPP) synthase (CrtE).

According to the in silico predictions, the cloned OS22 gene cluster appeared to be a ready-to-use CRT cassette. The role of two of these genes in carotenoid synthesis was unambiguously confirmed by transposon mutagenesis of the OS22 strain (see Methods for details). This experimental approach resulted in five mutant clones defective in pigment production (as judged from their white colonies), in which the transposon cassette had inserted into the *crtI* (4 mutants) or *crtB* (1 mutant) genes (Fig. 1). Further analysis was focused on revealing the transcriptional organization of the *crt locus*. We detected significant promoter activity within two tested DNA regions: upstream of the *crtE* gene (weaker promoter activity) and preceding the whole *crtWZYIB* gene array (stronger activity) (Fig. 1 and Additional file 1: Fig. S3).

### In vivo creation of plasmid pCRT01 and construction of carotenoid-producing strains

The activity of the CRT cassette was tested in *P. aminophilus* JCM 7686R (Rif^r^). Although plasmid pABW1-crt was unable to replicate in *Paracoccus* spp. it was introduced into JCM 7686R cells as a suicide plasmid by triparental mating, with the expectation that it would integrate with the host genome by means of illegitimate recombination (in our previous study we showed that illegitimate recombination is a common feature in *Paracoccus* spp. [32]). Indeed, two Km^r^ Rif^r^ transconjugant colonies were obtained, both with an intense orange color. Further analysis revealed that pABW1-crt had formed a stable cointegrate with natural plasmid pAMI2 (18.6 kb), which led to the creation of a shuttle replicon pCRT01 (Fig. 1). After introducing pCRT01 into *E. coli* DH5α by transformation, this replicon was transferred by triparental mating into *P. aminophilus* JCM 7686R and into another Rif^r^ recipient strain *P. kondratievae* NCIBM 13773R. pCRT01 initiated pigment synthesis in transconjugants of both strains, which suggested that this in vivo-created replicon may serve as a genetic tool for the construction of carotenoid-producing strains. The obtained strains were named *P. aminophilus* CRT1 and *P. kondratievae* CRT2.

DNA sequencing revealed that pCRT01 was created as a result of replicative transposition of the insertion sequence IS*Pam4* (IS*427* group of IS*5* family, that is present in pAMI2) into pABW1-crt (Fig. 1 and Additional file 2: Table S2). The resulting plasmid was very stably maintained in *P. aminophilus* CRT1 and *P. kondratievae* CRT2 cells: its loss was not detected after approx. 120 generations of growth in LB medium without antibiotic selection. This stability is the consequence of harboring of three efficient stabilization systems–toxin-antitoxin (TA), partition (PAR) and multimer resolution (MRS) systems–that have been shown to ensure plasmid maintenance in various *Alphaproteobacteria* [25].

**Fig. 3:**
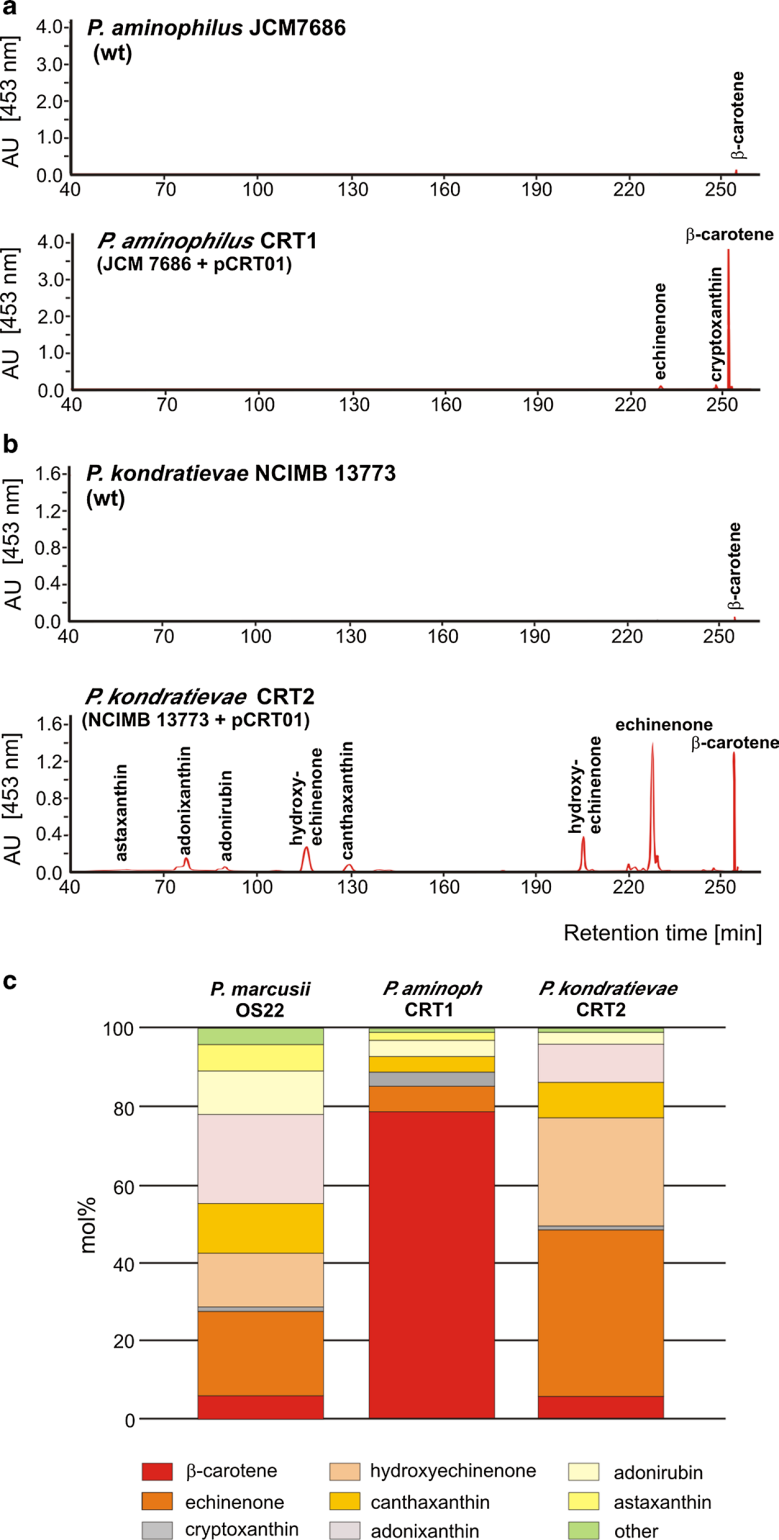
Identification of carotenoids produced by *P. aminophilus* CRT1 and *P. kondratievae* CRT2. **a**, **b**. Qualitative UPLC analysis of carotenoids of *P. aminophilus P. kondratievae* strains with and without plasmid pCRT01. The individual peaks on the chromatograms are annotated with the retention time and carotenoid identity. Only peaks for carotenoids produced at the level above 3.1 mol % (strain CRT1) or 4.2 mol % (CRT2) are visible. The peaks with different retention time (but with the same mass-to-charge, m/z, value) represent isomeric forms of the individual carotenoid species. C. Profiles of carotenoids produced by *P. marcusii* OS22, *P. aminophilus* CRT1 and *P. kondratievae* CRT2

The obtained CRT strains were then subjected to qualitative analysis of the pigments they produced using UPLC. Interestingly, this analysis revealed that each strain synthesized mixtures of carotenoids that differed in their composition. *P. aminophilus* CRT1 produced mainly β-carotene, while *P. kondratievae* CRT2 was able to produce various carotenoids, representing both the xanthophylls (astaxanthin, adonixanthin, adonirubin and canthaxanthin) and carotenes (echinenone, hydroxyechinenone and β-carotene) (Fig. 3a, b). Quantitative analysis confirmed statistically significant differences in profiles of biosynthesized carotenoids at the species levels (among *P. aminophilus* CRT1, *P. kondratievae* CRT2 and *P. marcusii* OS22) and indicated that β-carotene is produced in higher concentration by the CRT1 strain (Fig. 3c and Additional file 2: Table S3).

### Growth kinetics of CRT strains on low-cost carbon sources

**Fig. 4:**
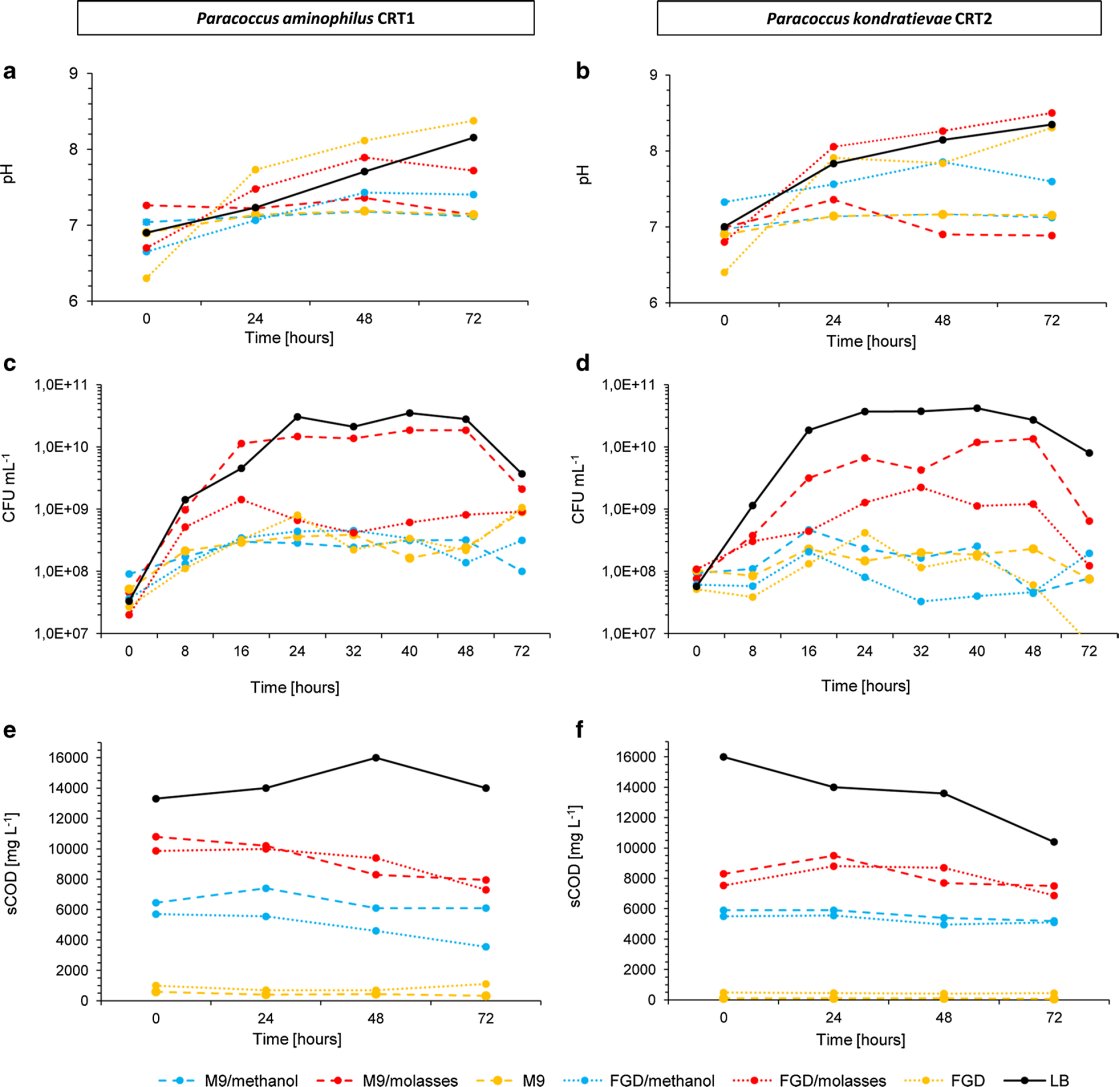
Characteristics of growth parameters of *Paracoccus* spp. producing carotenoids. Time-course profiles of growth (**a**, **b**), pH (**c**, **d**) and sCOD (**e**, **f**) in cultures of *P. aminophilus* CRT1 (**a**, **c**, **e**) and *P. kondratievae* CRT2 (**b**, **d**, **f**) grown on various media: LB, FGD, FGD/molasses (1%), FGD/methanol (0.5%), M9, M9/molasses (1%) and M9/methanol (0.5%). FGD–flue gas desulfurization effluent, LB–Lysogeny Broth. CFU mL^−1^–colony-forming units per milliliter, sCOD–soluble Chemical Oxygen Demand. All results are average of triplicate of three series of independent experiments. Standard deviations are shown in Additional file 2: (Table S5)

To develop a low-cost medium for the cultivation of *P. aminophilus* CRT1 and *P. kondratievae* CRT2, we examined the growth of these strains on various carbon sources, i.e. methanol and beet molasses (an industrial by-product rich in sucrose). The influence of both carbon sources on bacterial growth and the promotion of carotenoid production was tested by supplementing M9 minimal medium that contains all the required microelements. In addition, raw industrial effluent from flue gas desulfurization (FGD) from a coal-fired power plant, which contains high concentrations of sulfates (840 mg L^−1^), nitrates (130 mg L^−1^), chlorides (~ 7 g L^−1^) and metals/metalloids (e.g. Fe, Ni, Se, Zn) (Additional file 2: Table S4), was used as the basis of a test growth medium. Due to its composition (high mineral content), the fact that coal combustion still contributes significantly to electrical power generation globally and the possibility of using waste heat generated at power plants, FGD effluent represents a valuable source of secondary resources that should be managed [26]. LB medium was chosen as a positive control variant for growth and carotenoid production. M9 and FGD without added carbon sources were used as further controls. Time-course profiles for the growth of the two CRT strains in the different media are shown in Fig. 4 (statistical analysis of the results is presented in Additional file 2: Table S5).

Both strains were able to grow on M9 medium supplemented with the low-cost organic substrates methanol and molasses (Fig. 4c, d). In most cultures, the exponential phase occurred during the first 24 h of incubation. In the culture of strain *P. aminophilus* CRT1 with molasses and for both strains, in the culture with FGD/methanol the logarithmic growth was delayed; in these cases it occurred within 48 h (Fig. 4a).

The highest growth rate (expressed as doubling time) was observed for the cultures on media supplemented with molasses. With M9/molasses the doubling time was 4 h 50 min for *P. aminophilus* CRT1 and 4 h 13 min for *P. kondratievae* CRT2. Similar results were obtained for FGD/molasses (4 h 54 min and 4 h 9 min, respectively). A stationary phase characterized by a slight decline in the number of viable cells (expressed as CFU mL^−1^) between 24 and 48 h of incubation was observed only for *P. kondratievae* cultured on LB, M9, M9/molasses and FGD (Fig. 4d). In all other cultures there was no apparent stationary phase and cell numbers started to decline after they had reached their peak. For the *P. kondratievae* cultures grown on LB, M9 and M9/molasses, this “death” phase occurred after 48 h of incubation, when the number of cells decreased exponentially (Fig. 4b). In the *P. aminophilus* cultures, cell lysis started after 24 h of incubation on FGD and M9/methanol, and after 48 h on M9/molasses, FGD/molasses and FGD/methanol (Fig. 4c). For *P. aminophilus* CRT1 the highest rate of CFU mL^−1^ decrease of 3.91%·1 h^−1^ and 3.78%·1 h^−1^ were observed in the FGD/methanol and M9/molasses culture, respectively, which was far higher than the rate of 1.23%·1 h^−1^ for the FGD/molasses culture. For *P. kondratievae* CRT2 the rate of decrease was similar in all cultures, being approximately 2%·1 h^−1^. Taken together these results showed that the highest growth rates were achieved when molasses was added as a carbon source to the mineral nutrient media (Fig. 4c, d).

### Metabolic activity of bacteria during growth on low-cost substrates

The metabolic activity of *P. aminophilus* CRT1 and *P. kondratievae* CRT2 was determined by measuring time-course profiles of pH, soluble chemical oxygen demand (sCOD) and the content of reducing sugars in the cultures (Fig. 4). During growth, bacteria decompose organic matter or secrete metabolites that can change the pH of the growth medium. A growth-related pH increase was observed in all media. However, due to the presence of phosphate buffer in M9, the pH fluctuations in these cultures did not exceed 0.5. For both strains, the highest increase in pH occurred in cultures grown on FGD without an additional carbon source (ΔpH > 1.9). In the case of FGD/molasses the ΔpH was > 1.1, which was similar to the LB cultures. In mock-inoculated media, without bacteria, the pH values remained constant (data not shown).

sCOD is a measurement of the total chemically oxidizable material, which indicates the energy contained in the sample. By following changes in the sCOD it is possible to determine the assimilability of carbon sources and their optimal level in a bacterial culture. Decomposition of organic matter by the tested *Paracoccus* strains causes the sCOD to decrease. The highest efficiency of decomposition was achieved in the M9/molasses cultures. The reduction in sCOD by the end of the culture period (72 h) amounted to 26% for *P. aminophilus* CRT1 and 21% for *P. kondratievae* CRT2. For variants with added methanol the sCOD reduction did not exceed 20% for *P. aminophilus* CRT1 and 12% for *P. kondratievae* CRT2. The amounts of oxygen required for the oxidation of organic compounds in the FGD/molasses cultures were similar to those for the M9/molasses cultures and decreased by 27% and 22% after 72 h of incubation for *P. aminophilus* CRT1 and *P. kondratievae* CRT2, respectively. No significant changes in sCOD were recorded in the control LB culture for *P. aminophilus* CRT1 as opposed to significant decrease in the LB culture in the case of *P. kondratievae* CRT2–35% (Fig. 4e, f). The high sCOD values and their slight decrease after incubation suggest that the concentration of molasses used in the cultures was much higher than optimal. Thus, in order to minimize costs without any loss of efficiency, the amount of carbon source added should be reduced.

We also determined the content of reducing sugars in the cultures of the tested *Paracoccus* strains. Due to the fact that the main carbon source in molasses is sucrose, it is converted by the bacteria into reducing sugars whose content may be determined using the Luff-Schoorl method (Table 1). After 72 h of incubation the greatest reduction in sugar content was achieved in the M9/molasses cultures (approx. 33% for *P. aminophilus* CRT1 and 79% for *P. kondratievae* CRT2). In comparison, this reduction did not exceed 17% in the FGD/molasses cultures.

**Table 1:** The content of reducing sugars in bacterial cultures

Medium	Concentration of reducing sugars [mg L^−1^]^a^			
	T0	T24	T48	T72
*P. aminophilus* CRT1				
M9/molasses	6.27 ± 0.05	5.35 ± 0.037	4.79 ± 0.14	4.20 ± 0.59
FGD/molasses	5.46 ± 0.45	5.53 ± 1.18	5.09 ± 0.18	4.53 ± 0.36
*P. kondratievae* CRT2				
M9/molasses	6.31 ± 0.36	5.09 ± 0.09	4.13 ± 0.69	1.35 ± 0.66
FGD/molasses	4.90 ± 0.37	5.09 ± 0.65	4.64 ± 0.63	4.64 ± 0.45

T0-T72 time of cultivation [h]

^a^Mean ± SD (Standard deviation of triplicate experiments)

### Efficiency of carotenoid production on low-cost carbon sources

The level of carotenoids was analyzed and quantified after 72 h of bacterial growth in the media containing low-cost carbon sources. The highest concentrations of these compounds were found in the cultures containing molasses. The values were similar for both strains when cultured in M9/molasses, with an average concentration of 88.87 ng mL^−1^, whereas in the FGD/molasses cultures the carotenoid concentration was considerably higher for *P. aminophilus* CRT1 (127.09 ng mL^−1^) than for *P*. *kondratievae* CRT2 (58.8 ng mL^−1^). The highest efficiency of carotenoid production was found in the FGD/molasses cultures–117.68 μg g^−1^ d.w. for *P. aminophilus* CRT1 and 60 μg g^−1^ d.w. for *P. kondratievae* CRT2 (Table 2). In comparison, pigment production by bacteria grown on media supplemented with methanol was much lower. Efficiencies of only 8 µg g^−1^ d.w. and 19.52 µg g^−1^ d.w of carotenoids were obtained in the M9/methanol cultures of *P. aminophilus* and *P. kondratievae,* respectively. The highest pigment production efficiencies were observed in the control cultures grown on LB medium − 452.5 μg g^−1^ d.w. (*P. aminophilus* CRT1) and 265.4 μg g^−1^ d.w. (*P. kondratievae* CRT2).

**Table 2:** The content of carotenoids in bacterial cultures

Medium	Concentration [ng mL^−1^]	Efficiency [µg g^−1^ d.w.]
*P. aminophilus* CRT1		
M9/methanol	1.28	8.00
M9/molasses	90.40	90.40
FGD/molasses	127.09	117.68
FGD/methanol	2.23	1.76
LB	186.53	452.5
*P. kondratievae* CRT2		
M9/methanol	1.171	19.52
M9/molasses	87.34	37.32
FGD/molasses	58.80	60.00
FGD/methanol	2.045	1.70
LB	114.11	265.4

## Discussion

Chemical synthesis is currently used for the large-scale production of carotenoids. This process is expensive, generates a large amount of toxic waste and the final compounds may not have all the desired biological activities. Therefore, research on the microbial production of carotenoid pigments with high antioxidant capacity and intense color is of a great importance.

Plasmid pCRT01, obtained in this study, which carries the *crt* gene cluster of *P. marcusii* OS22 (CRT cassette), was found to be a convenient tool for the construction of bacterial strains that efficiently synthesize carotenoids. Two strains of biotechnological value were obtained using pCRT01: *P. aminophilus* CRT1–a β-carotene producer, and *P. kondratievae* CRT2, synthesizing a mixture of carotenoids (mainly xanthophylls). Thus, the introduction of the same gene cassette into different strains resulted in the synthesis of a different panel of pigments. This unexpected observation indicates the importance of the genetic and metabolic background of host strains when “implanting” heterologous metabolic pathways. It is noteworthy that the strains do not contain their own genes homologous to any of the genes of the *crtWZYIBE* cluster, which excludes possibility that the observed phenotypes were the result of expression of homologous and heterologous genes of varying catalytic activities.

Rather than introduce numerous genetic modifications that potentially alter the course of the carotenoid biosynthetic pathway, the novel methodological approach of this study was to use a single genetic cassette and generate efficient carotenoid producing strains through the selection of a suitable bacterial host. To confirm this strategy, pCRT01 should be introduced into a much larger pool of strains representing different taxonomic groups of *Alphaproteobacteria*, and the pigments produced by the resulting strains subjected to qualitative and quantitative analyses. These are immediate goals of our future studies.

The biosynthesis of carotenoids is a highly complex process. Its first steps result in the formation of a universal precursor–isopentenyl pyrophosphate (IPP) and its isomer, dimethylallyl pyrophosphate (DMAPP)–via the mevalonate (MVA) pathway of isoprenoid biosynthesis. The final stages of the pathway, associated with the modification of the synthesized molecules, generate the different carotenoid pigments (Fig. 2). Plasmid pCRT01 does not contain genes involved in the initial synthesis of IPP/DMAPP, which might limit its use. On the other hand, this may be a desirable feature, since the plasmid could serve as a sensitive probe that enables the identification of strains encoding a functional MVA pathway, which therefore produce natural precursors necessary for the synthesis of carotenoids. Such strains may potentially be better adapted to the production and accumulation of pigments because of the minimized metabolic burden and diminished probability of toxic effects exerted by overproduced compounds.

Plasmid pCRT01 was created by a fortuitous natural recombination event that led to the formation of a plasmid cointegrate composed of an *E. coli*-specific CRT-containing vector and a natural plasmid, pAMI2 of *P. aminophilus* JCM 6786. The *E. coli*-*Alphaproteobacteria* shuttle plasmid obtained in this manner shows high structural and segregational stability. Importantly, pCRT01 is a mobilizable replicon (contains a MOB module originating from broad-host-range plasmid RK2) so it can be readily transferred in a safe restriction-free way from *E. coli* into recipient cells in the presence of a helper conjugative system.

The alphaproteobacterial component of pCRT01, pAMI2, was previously analyzed in detail and found to contain (i) a replication system (REP) functional in bacteria representing all tested genera of *Alphaproteobacteria*, including *Agrobacterium*, *Brevundimonas*, *Ochrobactrum*, *Paracoccus*, *Rhizobium* and *Sinorhizobium* [27], (ii) an operon coding for an enzyme catalyzing the first step in the degradation of the toxic anthropogenic compound *N*,*N*-dimethylformamide (DMF), which enables methylotrophic growth of the host strain on DMF [28], and (iii) three types of plasmid stabilization system, including a *tad*-*ata* toxin-antitoxin (TA) system, responsible for efficient post-segregational elimination of plasmid-less cells from a bacterial population [25]. These stabilization systems ensure very stable plasmid maintenance in diverse alphaproteobacterial hosts [27], as was the case for pCRT01. The spontaneous loss of pCRT01 from *Paracoccus* spp. cells was not detected in non-selective conditions, which means that strains carrying the plasmid can be propagated in antibiotic-free media. This is an extremely important and desirable feature when considering large scale biotechnological applications.

The *crt* genes are usually present as a single copy in bacterial chromosomes, e.g. in *P. marcusii* OS22. Therefore, the plasmid localization of the CRT cassette ensures an increased gene dosage that may potentially result in more efficient production of carotenoids.

Currently, the microbial production of carotenoids cannot compete economically with chemical synthesis [29]. One reason for this is the high cost of biomass production, which leads to a low efficiency of pigment recovery. The use of cheap mineral nutrient and carbon sources may significantly reduce the cost of obtaining these bioactive compounds. In this study we developed a low-cost medium based on industrial waste materials. Through examining the growth rate, changing pH, sCOD values and reduction in the level of reducing sugars we identified flue gas desulfurization waste supplemented with molasses as a medium that supports efficient growth of carotenoid-producing bacteria.

Molasses is a very abundant industrial by-product that is commonly used in many biotechnological applications, including biogas and ethanol production [30, 31]. The nutrient load included in the molasses (approximately 50% sucrose plus crude proteins, calcium, phosphorus, potassium, sodium, chlorine and sulfur) accelerated the increase in bacterial biomass and proved to be a good source of carbon for the carotenoid-producing *Paracoccus* strains. Furthermore, efficient bacterial growth and high concentrations of carotenoids were observed when the medium contained FGD waste (originating from a coal-fired power plant located in central Poland). Therefore, it may be concluded that FGD effluent (despite its high sulfate and metals content) can effectively replace expensive synthetic media in large-scale production processes. However, it should be noted that FGD may be burdened with increased contents of metals and inorganic anions (mainly sulfates), therefore industrial application requires bio-process optimization and verification of the quality of produced carotenoid dyes.

Optimization strategies described in this study were orientated into increasing biomass production as this may strictly correlate with an increased carotenoids production. However, this approach and our findings may be extrapolated for optimization of the production of any other metabolite using *Paracoccus* strains as microbial cell factories.

## Conclusion

With the aim of optimizing the microbial bioproduction of carotenoids, the presented multi-stage experimental approach included the isolation of a CRT cassette, the generation of an appropriate genetic tool (plasmid pCRT01) and its application in the construction of carotenoid-producing strains, plus the development of media based on low-cost waste materials for the efficient growth of these bacteria. An important environmental aspect of this study is the utilization of industrial wastes. Our future research will be focused on optimizing the bacterial culture conditions and testing the pigment-producing systems (strains and media) at a larger biotechnological scale in order to produce microbial carotenoids in a cost-effective manner.

## Methods

### Bacterial strains and culture conditions

The following bacterial strains were used in this study: *P. marcusii* OS22 [21], *P. aminophilus* JCM 7686R (rifampicin resistant, Rif^r^ derivative of strain JCM 7686) [28], *P. kondratievae* NCIBM 131773R (Rif^r^ derivative of strain NCIMB 13773^T^) [32], *Paracoccus versutus* UW225 [33], *E. coli* TG1 [34], *E. coli* DH5α [34], *E. coli* S17-1 [35] and DH5αpir [36]. Characteristics of the strains used and constructed in this study are presented in Additional file 2: (Table S6). All strains were grown in Lysogeny Broth (LB) (10 g L^−1^ tryptone, 5 g L^−1^ yeast extract, 5 g L^−1^ NaCl) [37] or M9 minimal medium (12.8 g L^−1^ Na_2_HPO_4_·7H_2_O, 3 g L^−1^ KH_2_PO_4_, 0.5 g L^−1^ NaCl, 1 g L^−1^ NH_4_Cl, 2 mM MgSO_4_, 0.1 mM CaCl_2_) [37] at 30 °C (*Paracoccus* spp.) and 37 °C (*E. coli* strains). Where necessary, the medium was supplemented with antibiotics: kanamycin (50 μg mL^−1^) and rifampicin (50 μg mL^−1^). Two strains constructed in this study, *P. aminophilus* CRT1 and *P. kondratievae* CRT2, were deposited in the IAFB Collection of Industrial Microorganisms of the Institute of Agricultural and Food Biotechnology in Warsaw (Poland) as *P. aminophilus* KKP 2053p and *P. kondratievae* KKP 2054p, respectively, on 10th February 2014.

### Batch assays of carotenoid production on low-cost substrates

The ability of *P. aminophilus* CRT1 and *P. kondratievae* CRT2 to grow on low-cost substrates was tested by inoculating M9 minimal medium or flue gas desulfurization wastewater (FGD) containing 0.5% methanol or 1% beet molasses as the sole carbon source. In control variants, bacterial strains were cultivated in LB medium, and in FGD and M9 medium without molasses. Fresh overnight cultures propagated in LB medium were centrifuged at 5000*g*, the pellets were rinsed and suspended in 0.8% NaCl solution, and used to inoculate 300 mL lots of the different media (in 500 ml flasks) to a final density of approximately 10^6^ cells mL^−1^ (OD_600_ ~ 0.08). The cultures were then grown aerobically at 30 °C for 72 h. Microbial growth was measured at 24 h intervals by plating diluted culture samples on agar-solidified LB medium to determine the number of colony-forming units per milliliter (CFU mL^−1^). The rate of growth (doubling time) was determined using the following equation:

$$Growth rate = \frac{{{\text{duration }} \times \log \left( 2 \right)}}{{\log \left( {final CFU ml^{ - 1} } \right) - \log \left( {initial CFU ml^{ - 1} } \right)}}$$

To monitor organic substrate degradation, soluble chemical oxygen demand (sCOD) was determined using a compact PF-12Plus photometer and the photo-metric tests NANOCOLOR^®^ COD 1500 and 160, and NANOCOLOR^®^ organic Acids 3000, as indicated by the manufacturer (Machery-Nagel GmbH, Düren, Germany). pH was measured using an Elmetron CPC-411 pH meter. The change in pH of the cultures (ΔpH) was calculated using the equation ΔpH = pH_end_ − pH_start_. To determine the content and quality of the produced carotenoids, biomass was collected after 72 h of culture. Culture volumes of 50 ml were centrifuged and the pellets dried at 105 °C for 24 h before weighing to determine the dry mass.

### Determination of reducing sugars content

The content of reducing sugars in the test cultures was determined after inversion according to standard PN-90 A-79120/06 (Luff-Schoorl method) [38]. The method is based on the reduction of Cu^2+^ in the Luff-Schoorl solution (copper citrate with sodium carbonate) to Cu^+^. The excess of Cu^2+^ remaining after the reaction was determined by the iodometric method. Briefly, sugars in the culture supernatants, obtained after centrifugation at 5000*g*, were hydrolyzed with hydrochloric acid at 68 °C for 5 min, then the samples were chilled to 20 °C and neutralized with 20% sodium hydroxide solution through titration using phenolophtalein as indicator, to slightly alkaline pH. To determine the concentration of reducing sugars the samples (25 mL) were mixed with Luff-Schoorls solution (25 mL) and boiled for 10 min. After cooling the sample tubes rapidly in a stream of cold water, 30% KI (10 mL) solution and 6 M H_2_SO_4_ (25 mL) were added. The liberated iodine was titrated with 0.1 M sodium thiosulphate and 0.5% starch indicator solution was added to identify the end point.

### Qualitative and quantitative analysis of carotenoids

The extraction of carotenoids was carried out in a climate-room at 4 °C, in dim light, as described earlier for plant pigments [39], with some modifications. The bacterial pellet was resuspended in 1.5 mL acetone-methanol (8:2 v/v), sonicated at ultrasonic cleaner for 5 min and perfused with argon for 2 min. Then hexane (4.5 mL) was added and the sample was shaking in a reciprocating shaker (PROMAX 2020, Heidolph, Germany) for 30 min in the dark. After shaking, the sample was incubated without agitation for 5 min to allow phase separation. The upper hexane phase was collected by aspiration and transferred to a 100 mL Erlenmeyer flask, perfused with argon, capped and stored in the dark in 4 °C. In the second extraction stage, 1 mL of propanol was used in addition to hexane, and perfusion, shaking and phase collection were repeated as before. After removal of hexane, the polar phase was centrifuged for 15 min at 4500 rpm (MPW-340 centrifuge, PL). The supernatant was combined with the two hexane phases, perfused with argon, and filtered through a Millipore syringe filter unit Millex-CV13 Filter Unit (0.22 μm). Then evaporated to dryness under argon, and dissolved in 1 mL methanol-propanol-hexane 6:1:3 (v/v/v). Dissolved samples were transferred to 2 mL glass vials and stored at − 80 °C.

Extracted pigments were separated using the Acquity Ultra Performance LC system (Waters) connected with the Synapt G2 HDMS mass spectrometer (Waters). The samples were injected into an Acquity UPLC HSS T3 (1.8 µm, 1.0 × 150 mm) analytical column and eluted by multistep linear gradient of solvent A (water: methanol 15:85, v/v) and B (methanol: 2-propanol: hexane 2:1:1, v/v).

The samples (7.5 μl) were injected into column and eluted at 25 °C at a constant flow rate of 35 μl min^−1^ with 100% of solvent A during 15 min. Next, the linear gradient of buffer B was distributed as follows: 0–15% B in the 15–160 min (flow rate 35 μl min^−1^); 15–80% B in 160–240 min (flow rate 35–20 μl min^−1^); 80–90% B in a 240–245 min (flow rate 20 μl min^−1^); 90–100% B in 245–255 min (flow rate 20–80 μl min^−1^) and hold for 10 min at 100% B. During the next 5 min concentration of solvent B was decreased to 0% and the column was equilibrated with solvent A for 15 min (flow rate 80–5 μl min^−1^) before the next injection. Separation of samples was monitored by a photodiode array detector at 200–700 nm range and a mass spectrometer at 100–1000 m/z range. The chromatograms were analyzed with MassLynx 4.1 software (Waters).

The quantitative estimation of carotenoids was carried out by the method of Liaaen-Jensen and Jensen [40]. The bacterial pellet was resuspended in 10 mL of acetone-methanol (7:2 v/v) sonicated at ultrasonic cleaner for 5 min and filtered through Whatman filter paper. The absorbance (O.D.) of this extracted solution was measured at 453 and 488 λ.

### Plasmids used and constructed in this study

The following plasmids were used in this study: (i) pABW1—cloning vector specific to *E. coli* (Km^r^, *ori* pMB1, *oriT* RK2) [41], (ii) pCM132–*lacZ* reporter gene fusion vector (Km^r^) [42], (iii) pRK2013–helper plasmid in conjugal transfer (Km^r^, *ori* ColE1, conjugal transfer genes of RK2) [43], (iv) pUToriγKm (Tn5-based delivery plasmid with Km^r^, *ori* R6K, MOB RK2, tnp* gene of Tn5-IS50R). Plasmid pABW1-crt was constructed in the following way. The *crtW* gene was amplified by PCR using the primers CRTWL and CRTWR (Additional file 2: Table S6), and *P. marcusii* OS22 genomic DNA as the template. The amplified gene fragment was digested using XbaI and inserted into the corresponding site in the vector pABW1 to create pABW1-crtW. A detailed scheme for the construction of pABW1-crt is shown in Additional file 1: (Fig. S2). Details of construction of pCM132 plasmid derivatives, used for the identification of promoters within the *crt* locus, are described in Additional file 2: (Table S6).

### Standard genetic manipulations

Common genetic manipulation procedures were performed according to standard protocols of Sambrook and Russell [37]. Plasmids of *Paracoccus* spp. were isolated using the method of Birnboim and Doly [45], and when required, the DNA was further purified by CsCl-ethidium bromide gradient centrifugation [37]. Plasmid DNA was isolated from *E. coli* cells using a Plasmid Mini Kit (A&A Biotechnology). Restriction endonucleases and T4 DNA ligase were used according to the supplier’s instructions (Thermo Scientific). Polymerase chain reaction (PCR) was carried out using Pfu or Phusion DNA polymerases (Thermo Scientific) following the manufacturer’s instructions. Amplifications using these thermostable DNA polymerases were performed in a Mastercycler (Eppendorf) with synthetic oligonucleotide primers and appropriate DNA templates. The PCR-amplified DNA fragments were analyzed by electrophoresis on 0.8% agarose gels and, where necessary, purified using a Gel-Out Kit (A&A Biotechnology). Chemical transformation of *E. coli* strains was performed according to the method of Kushner [46]. Triparental mating to introduce plasmids into *Paracoccus* spp. cells was performed as described previously [47].

### Plasmid stability assay

The stability of pCRT01 in *Paracoccus* spp. strains was tested during growth under nonselective conditions as described previously [25]. Briefly, stationary-phase cultures were diluted in fresh medium without antibiotic selection and cultivated for approximately 120 generations. Samples were diluted and plated onto solid medium in the absence of selective antibiotics. One hundred of the resulting colonies were tested for the presence of the plasmid-encoded Km^r^ marker by replica plating. The percentage of kanamycin resistant colonies was use as a measure of plasmid retention.

### Detection of gene promoter activity within the *crt* locus

To identify *crt* gene promoters, DNA regions upstream of the individual genes were amplified by PCR and inserted into broad-host-range promoter probe vector pCM132 to generate transcriptional fusions with a promoter-less *lacZ* reporter gene (oligonucleotide primers used are shown in Additional file 2: Table S6). The activity of the putative promoters was examined in *P. versutus* UW225 (routinely used as a host strain for *Paracoccus* plasmids) using a microtiter plate assay for β-galactosidase activity [48]. A strain carrying the “empty” (promoter-less) vector pCM132 was used as a negative control. Appearance of the yellow product was monitored using a Tecan Sunrise plate reader (absorbance at 415 nm). The β-galactosidase activity in Miller units was calculated from the slopes of the kinetic plots as described previously [48].

### Transposon mutagenesis

Transposon mutants of *P. marcusii* OS22 were generated using suicide plasmid pUToriγKm (derivative of pUTKm [44] containing a novel Km^r^ cassette from plasmid pDIY-KM [27] and origin of replication, *oriγ*, of plasmid R6K). The plasmid was transferred from *E. coli* into OS22 cells by triparental mating and transconjugants were selected on LB agar medium containing kanamycin and rifampicin. Genomic DNAs of mutants defective in pigment production were isolated, digested with appropriate restriction endonucleases and treated with T4 DNA ligase (Stratagene). Following rescue of the transposon-containing plasmids in *E. coli* S17-1 (encodes replication initiation protein that activates *ori𝛾*) the transposon insertion sites were determined by sequencing with oligonucleotide primers FPUT and RPUT (Additional file 2: Table S6).

### DNA sequencing and bioinformatic analysis

The complete nucleotide sequence of plasmid pCRT01 was determined by the DNA Sequencing and Oligonucleotide Synthesis Laboratory (oligo.pl) at the Institute of Biochemistry and Biophysics, Polish Academy of Sciences, Warsaw. A MID-tagged shotgun plasmid-library was sequenced using a FLX Titanium Genome Sequencer (Roche/454 Life Sciences). Newbler de novo assembler software (Roche) was used for the sequence assembly. Final gap closure and sequence polishing were performed by capillary sequencing of PCR products using an ABI3730xl DNA Analyzer (Applied Biosystems). The plasmid nucleotide sequence was analyzed using Clone Manager (Sci-Ed8) and Artemis software [49]. Similarity searches were performed using the BLAST programs [50] provided by the National Center for Biotechnology Information (NCBI) (http://blast.ncbi.nlm.nih.gov/Blast.cgi).

### Nucleotide sequence accession number

The nucleotide sequence of the *P. marcusii* OS22 *crt* gene cluster was deposited in GenBank (NCBI) with the accession number MT175370.

## Supplementary information

**Additional file 1: Figure S1.** Qualitative UPLC analysis of carotenoids produced by *P. marcusii* OS22. **Figure S2.** Scheme of construction of plasmid pABW1-crt containing the *crt* genes of *P. marcusii* OS22. **Figure S3.** Identification of promoters within the *crt* locus of *P. marcusii* OS22.

**Additional file 2: Table S1.** Absorbance and ESI mass spectrometry data for carotenoids produced by *P. marcusii* OS22. **Table S2.** Predicted genes of plasmid pCRT01. **Table S3.** Quantitative analysis of carotenoids identified in extracts of *Paracoccus* spp. cells. **Table S4.** The content of anions and elements in flue gas desulfurization (FGD) wastewater. **Table S5.** Standard deviation (%) of growth parameters of *Paracoccus* spp. producing carotenoids. **Table S6.** Bacterial strains, plasmids and oligonucleotides used in this study.

## Data Availability

All data generated or analysed during this study are included in this published article [and its Additional files].

## Abbreviations

CrtB: Phytoene synthase
CrtE: Geranylgeranyl diphosphate synthase
CrtW: β-carotene oxygenase
CrtY: Lycopene cyclase
CrtZ: β-carotene hydroxylase
DMAPP: Dimethylallyl pyrophosphate
DMF: *N*,*N*-dimethylformamide
FGD: Flue gas desulfurization
GGPP: Geranylgeranyl diphosphate
IPP: Isopentenyl pyrophosphate
MVA: Mevalonate
sCOD: Soluble chemical oxygen demand
UPLC: Ultra-performance liquid chromatography

## References

[1] Kushwaha K, Saini A, Saraswat P, Agarwal MK, Saxena J (2014). Colorful world of microbes: carotenoids and their applications. Adv Biol..

[2] Hashimoto H, Uragami C, Cogdell RJ (2016). Carotenoids and photosynthesis. Subcell Biochem.

[3] Avalos J, Carmen Limon M (2015). Biological roles of fungal carotenoids. Curr Genet.

[4] Mathews MM, Sistrom WR (1959). Function of carotenoid pigments in non-photosynthetic bacteria. Nature.

[5] Handelman GJ, Dratz EA, Reay CC, van Kuijk JG (1988). Carotenoids in the human macula and whole retina. Invest Ophthalmol Vis Sci.

[6] Bhosale P, Bernstein PS (2005). Synergistic effects of zeaxanthin and its binding protein in the prevention of lipid membrane oxidation. Biochim Biophys Acta.

[7] Higuera-Ciapara I, Felix-Valenzuela L, Goycoolea FM (2006). Astaxanthin: a review of its chemistry and applications. Crit Rev Food Sci Nutr.

[8] MacWilliams A. The global market of carotenoids. BCC Research. 2018:FOD025F.

[9] Barreiro C, Barredo JL (2018). Carotenoids production: a healthy and profitable industry. Methods Mol Biol.

[10] Wang C, Zhao S, Shao X, Park JB, Jeong SH, Park HJ, Kwak WJ, Wei G, Kim SW (2019). Challenges and tackles in metabolic engineering for microbial production of carotenoids. Microb Cell Fact.

[11] Ye L, Zhang C, Bi C, Li Q, Zhang X (2016). Combinatory optimization of chromosomal integrated mevalonate pathway for beta-carotene production in *Escherichia coli*. Microb Cell Fact.

[12] Yang JM, Guo LZ (2014). Biosynthesis of beta-carotene in engineered *E. coli* using the MEP and MVA pathways. Microb Cell Fact..

[13] Schweiggert RM, Carle R, Kaczor A (2016). Carotenoid production by bacteria, microalgae, and fungi. Carotenoids: nutrition, analysis and technology.

[14] Kelly DP, Rainey FA, Wood AP. The Genus *Paracoccus*. Prokaryotes: A Handbook on the Biology of Bacteria, Vol 5, Third Edition. 2006:232–49.

[15] Harker M, Hirschberg J, Oren A (1998). *Paracoccus marcusii* sp. nov., an orange gram-negative coccus. Int J Syst Bacteriol..

[16] Tsubokura A, Yoneda H, Mizuta H (1999). *Paracoccus carotinifaciens* sp. nov., a new aerobic gram-negative astaxanthin-producing bacterium. Int J Syst Bacteriol..

[17] Lee JH, Kim YT (2006). Cloning and characterization of the astaxanthin biosynthesis gene cluster from the marine bacterium *Paracoccus haeundaensis*. Gene.

[18] Urakami T, Araki H, Oyanagi H, Suzuki K, Komagata K (1990). *Paracoccus aminophilus* sp. nov. and *Paracoccus aminovorans* sp. nov., which utilize N, N-dimethylformamide. Int J Syst Bacteriol..

[19] Dziewit L, Czarnecki J, Wibberg D, Radlinska M, Mrozek P, Szymczak M, Schluter A, Puhler A, Bartosik D (2014). Architecture and functions of a multipartite genome of the methylotrophic bacterium *Paracoccus aminophilus* JCM 7686, containing primary and secondary chromids. BMC Genomics..

[20] Doronina NV, Trotsenko YA, Kuznetzov BB, Tourova TP (2002). Emended description of *Paracoccus kondratievae*. Int J Syst Evol Microbiol.

[21] Drewniak L, Styczek A, Majder-Lopatka M, Sklodowska A (2008). Bacteria, hypertolerant to arsenic in the rocks of an ancient gold mine, and their potential role in dissemination of arsenic pollution. Environ Pollut.

[22] Misawa N, Satomi Y, Kondo K, Yokoyama A, Kajiwara S, Saito T, Ohtani T, Miki W (1995). Structure and functional analysis of a marine bacterial carotenoid biosynthesis gene cluster and astaxanthin biosynthetic pathway proposed at the gene level. J Bacteriol.

[23] Karczewska-Golec J, Kochanowska-Lyzen M, Balut M, Piotrowski A, Golec P, Szalewska-Palasz A (2019). Draft genome sequence of *Paracoccus* sp. strain 228, isolated from surface water of the Gulf of Gdansk in the Baltic Sea. Microbiol Resour Announc..

[24] Machado H, Sonnenschein EC, Melchiorsen J, Gram L (2015). Genome mining reveals unlocked bioactive potential of marine Gram-negative bacteria. BMC Genomics..

[25] Dziewit L, Jazurek M, Drewniak L, Baj J, Bartosik D (2007). The SXT conjugative element and linear prophage N15 encode toxin-antitoxin-stabilizing systems homologous to the *tad*-*ata* module of the *Paracoccus aminophilus* plasmid pAMI2. J Bacteriol.

[26] Cordoba P, Staicu LC (2018). Flue gas desulfurization effluents: an unexploited selenium resource. Fuel.

[27] Dziewit L, Adamczuk M, Szuplewska M, Bartosik D (2011). DIY series of genetic cassettes useful in construction of versatile vectors specific for *Alphaproteobacteria*. J Microbiol Methods.

[28] Dziewit L, Dmowski M, Baj J, Bartosik D (2010). Plasmid pAMI2 of Paracoccus aminophilus JCM 7686 carries N, N-dimethylformamide degradation-related genes whose expression is activated by a LuxR family regulator. Appl Environ Microbiol.

[29] Schmidt I, Schewe H, Gassel S, Jin C, Buckingham J, Humbelin M, Sandmann G, Schrader J (2011). Biotechnological production of astaxanthin with *Phaffia rhodozyma*/*Xanthophyllomyces dendrorhous*. Appl Microbiol Biotechnol.

[30] Detman A, Chojnacka A, Blaszczyk M, Kazmierczak W, Piotrowski J, Sikora A (2017). Biohydrogen and biomethane (biogas) production in the consecutive stages of anaerobic digestion of molasses. Pol J Environ Stud..

[31] Lee JY, Yun J, Kim TG, Wee D, Cho KS (2014). Two-stage biogas production by co-digesting molasses wastewater and sewage sludge. Bioprocess Biosyst Eng.

[32] Dziewit L, Baj J, Szuplewska M, Maj A, Tabin M, Czyzkowska A, Skrzypczyk G, Adamczuk M, Sitarek T, Stawinski P (2012). Insights into the transposable mobilome of *Paracoccus* spp. (*Alphaproteobacteria*). PLoS ONE..

[33] Bartosik D, Baj J, Plasota M, Piechucka E, Wlodarczyk M (1993). Analysis of *Thiobacillus versutus* pTAV1 plasmid functions. Acta Microbiol Polon..

[34] Gibson TJ. Studies on Epstein-Barr genome. PhD thesis, University of Cambridge. 1984.

[35] Priefer UB, Simon R, Puhler A (1985). Extension of the host range of *Escherichia coli* vectors by incorporation of RSF1010 replication and mobilization functions. J Bacteriol.

[36] Platt R, Drescher C, Park SK, Phillips GJ (2000). Genetic system for reversible integration of DNA constructs and *lacZ* gene fusions into the *Escherichia coli* chromosome. Plasmid.

[37] Sambrook J, Russell DW (2001). Molecular cloning: a laboratory manual.

[38] Egan H, Kirk R, Sawyer R: The Luff Schoorl Method. Sugars and preserves. In: Pearson’s Chemical Analysis of Foods. 8th edn. Harlow, UK: Longman Scientific and Technical; 1981: 152–3.

[39] Szalonek M, Sierpien B, Rymaszewski W, Gieczewska K, Garstka M, Lichocka M, Sass L, Paul K, Vass I, Vankova R (2015). Potato annexin STANN1 promotes drought tolerance and mitigates light stress in transgenic *Solanum tuberosum* L. Plants. PLoS ONE..

[40] Liaaen-Jensen S, Jensen A: Quantitative determination of carotenoids in photosynthetic tissues. In: Methods in Enzymology. Academic Press; 1971:586–602.

[41] Bartosik D, Bialkowska A, Baj J, Wlodarczyk M (1997). Construction of mobilizable cloning vectors derived from pBGS18 and their application for analysis of replicator region of a pTAV202 mini-derivative of *Paracoccus versutus* pTAV1 plasmid. Acta Microbiol Pol.

[42] Marx CJ, Lidstrom ME (2001). Development of improved versatile broad-host-range vectors for use in methylotrophs and other Gram-negative bacteria. Microbiology.

[43] Ditta G, Stanfield S, Corbin D, Helinski DR (1980). Broad host range DNA cloning system for gram-negative bacteria: construction of a gene bank of *Rhizobium meliloti*. Proc Natl Acad Sci USA..

[44] de Lorenzo V, Herrero M, Jakubzik U, Timmis KN (1990). Mini-Tn5 transposon derivatives for insertion mutagenesis, promoter probing, and chromosomal insertion of cloned DNA in gram-negative eubacteria. J Bacteriol.

[45] Birnboim HC, Doly J (1979). A rapid alkaline extraction procedure for screening recombinant plasmid DNA. Nucleic Acids Res.

[46] Kushner SR: An improved method for transformation of *E. coli* with ColE1 derived plasmids. In: Genetic Engineering. Edited by Boyer HB, Nicosia S; 1978:17–23.

[47] Bartosik D, Szymanik M, Wysocka E (2001). Identification of the partitioning site within the *repABC*-type replicon of the composite *Paracoccus versutus* plasmid pTAV1. J Bacteriol.

[48] Thibodeau SA, Fang R, Joung JK (2004). High-throughput beta-galactosidase assay for bacterial cell-based reporter systems. Biotechniques.

[49] Carver T, Berriman M, Tivey A, Patel C, Bohme U, Barrell BG, Parkhill J, Rajandream MA (2008). Artemis and ACT: viewing, annotating and comparing sequences stored in a relational database. Bioinformatics.

[50] Altschul SF, Madden TL, Schaffer AA, Zhang J, Zhang Z, Miller W, Lipman DJ (1997). Gapped BLAST and PSI-BLAST: a new generation of protein database search programs. Nucleic Acids Res.

