# Supplementary material for: In vivo creation of plasmid pCRT01 and its use for the construction of carotenoid-producing Paracoccus spp. strains that grow efficiently on industrial wastes
Source: Microb Cell Fact. 2020 Jul 13;19:141. doi: 10.1186/s12934-020-01396-z (PMC7359593; doi:10.1186/s12934-020-01396-z)
Supplement: Supplementary file 1 — Additional file 1: Figure S1. Qualitative UPLC analysis of carotenoids produced by P. marcusii OS22. Figure S2. Scheme of construction of plasmid pABW1-crt containing the crt genes of P. marcusii OS22. Figure S3. Identification of promoters within the crt locus of P. marcusii OS22. [file 12934_2020_1396_MOESM1_ESM.pdf]

Additional Materials (file 1)
(Figures S1-S3)

In vivo creation of plasmid pCRT01 and its use for the construction of carotenoid-producing *Paracoccus* spp. strains that grow efficiently on industrial wastes

Anna Maj[1], Lukasz Dziewit[2], Lukasz Drewniak[2], Maciej Garstka[3], Tomasz Krucon[2], Katarzyna Piatkowska[2], Katarzyna Gieczewska[4], Jakub Czarnecki[1,5], Ewa Furmanczyk[1,6], Robert Lasek[1], Jadwiga Baj[1], Dariusz Bartosik[1*]

[1]Department of Bacterial Genetics, Institute of Microbiology, Faculty of Biology, University of Warsaw, Miecznikowa 1, 02-096 Warsaw, Poland; aniaklicka@poczta.fm (AM), jczarnecki@biol.uw.edu.pl (JC), e_furmanczyk@poczta.fm (EF), lasek@biol.uw.edu.pl (RL), bajja@biol.uw.edu.pl (JB), bartosik@biol.uw.edu.pl (DB)

[2]Department of Environmental Microbiology and Biotechnology, Institute of Microbiology, Faculty of Biology, University of Warsaw, Miecznikowa 1, 02-096 Warsaw, Poland; ldziewit@biol.uw.edu.pl (LDz), ldrewniak@biol.uw.edu.pl (LDr), tkrucon@biol.uw.edu.pl (TK), kpiatkowska@biol.uw.edu.pl (KP)

[3]Department of Metabolic Regulation, Institute of Biochemistry, Faculty of Biology, University of Warsaw, Miecznikowa 1, 02-096 Warsaw, Poland; garstka@biol.uw.edu.pl (MG)

[4]Department of Plant Anatomy and Cytology, Institute of Experimental Plant Biology and Biotechnology, Faculty of Biology, University of Warsaw, Miecznikowa 1, 02-096 Warsaw, Poland; kat.gieczewska@biol.uw.edu.pl (KG)

[5]Bacterial Genome Plasticity, Department of Genomes and Genetics, Institut Pasteur, Paris, France

[6]Department of Plant Protection from Pests, Research Institute of Horticulture, Skierniewice, Poland

***Corresponding author:** bartosik@biol.uw.edu.pl

Department of Bacterial Genetics, Institute of Microbiology, Faculty of Biology, University of Warsaw, Miecznikowa 1, 02-096 Warsaw, Poland

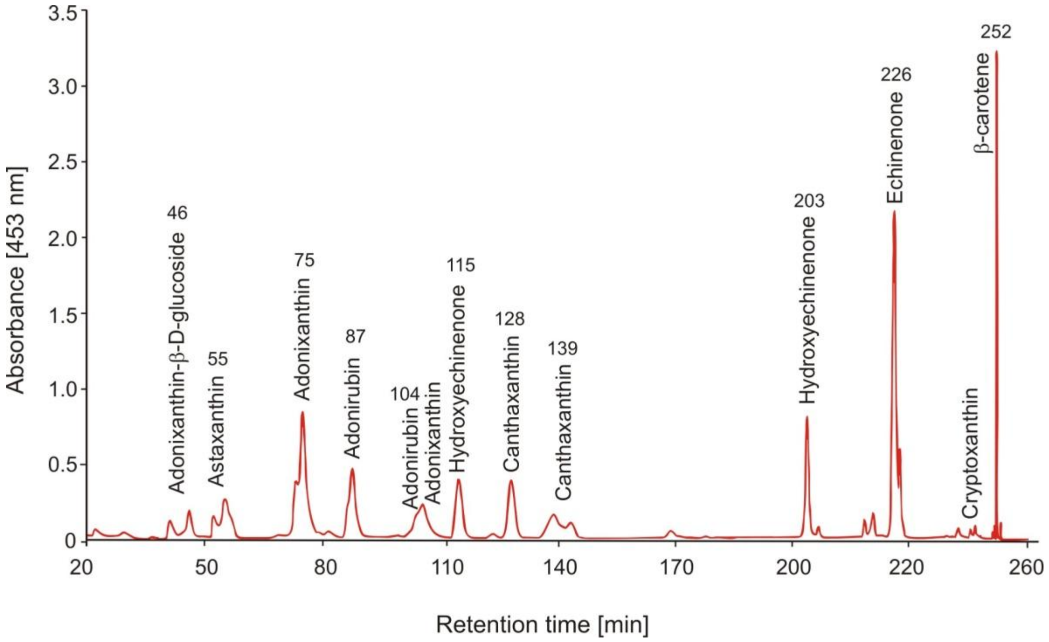

Figure S1. Qualitative UPLC analysis of carotenoids produced by *P. marcusii* OS22.

The individual peaks on the chromatograms are annotated with the retention time and carotenoid identity. The peaks with different retention time (but with the same mass-to-charge, m/z, value) represent isomeric forms of the individual carotenoid species.

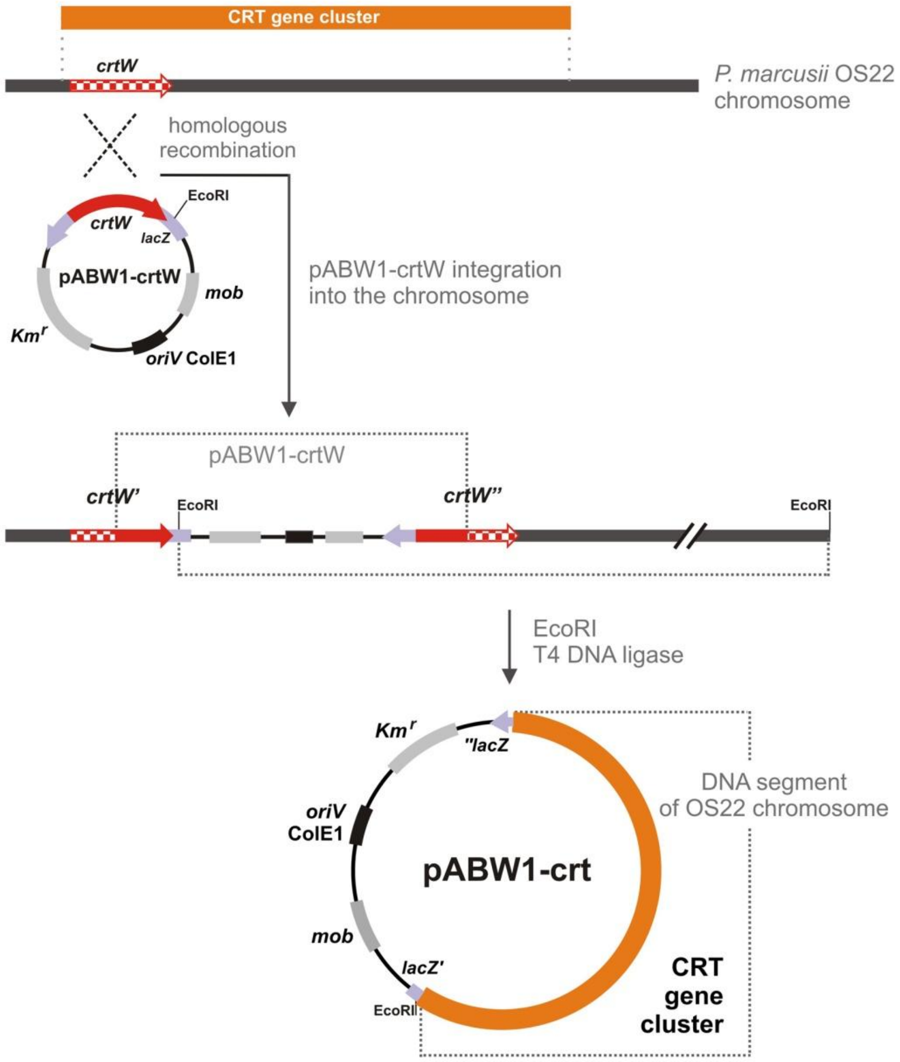

Figure S2. Scheme of construction of plasmid pABW1-crt containing the *crt* genes of *P. marcusii* OS22.

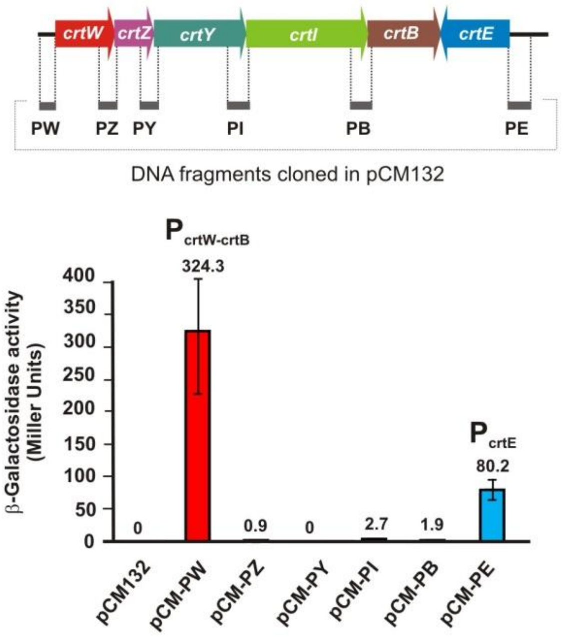

Figure S3. Identification of promoters within the *crt* locus of *P. marcusii* OS22.

pCM132 - promoter probe vector. pCM- plasmids were constructed by amplification by PCR of DNA regions upstream of the individual *crt* genes and insertion of the amplified fragments into pCM132 to generate transcriptional fusions with a promoter-less *lacZ* reporter gene (see Additional file 2: Table S6 for details on the plasmids construction). β-Galactosidase activity (tested in *Paracoccus versutus* UW225) reflected the presence and strength of the cloned promoters.
