# Supplementary material for: In vivo creation of plasmid pCRT01 and its use for the construction of carotenoid-producing Paracoccus spp. strains that grow efficiently on industrial wastes
Source: Microb Cell Fact. 2020 Jul 13;19:141. doi: 10.1186/s12934-020-01396-z (PMC7359593; doi:10.1186/s12934-020-01396-z)
Supplement: Supplementary file 2 — Additional file 2: Table S1. Absorbance and ESI mass spectrometry data for carotenoids produced by P. marcusii OS22. Table S2. Predicted genes of plasmid pCRT01. Table S3. Quantitative analysis of carotenoids identified in extracts of Paracoccus spp. cells. Table S4. The content of anions and elements in flue gas desulfurization (FGD) wastewater. Table S5. Standard deviation (%) of growth parameters of Paracoccus spp. producing carotenoids. Table S6. Bacterial strains, plasmids and oligonucleotides used in this study. [file 12934_2020_1396_MOESM2_ESM.pdf]

# Additional Materials (file 2)
## (Tables S1-S6)

# *In vivo* creation of plasmid pCRT01 and its use for the construction of carotenoid-producing *Paracoccus* spp. strains that grow efficiently on industrial wastes

Anna Maj[1], Lukasz Dziewit[2], Lukasz Drewniak[2], Maciej Garstka[3], Tomasz Krucon[2], Katarzyna Piatkowska[2], Katarzyna Gieczewska[4], Jakub Czarnecki[1,5], Ewa Furmanczyk[1,6], Robert Lasek[1], Jadwiga Baj[1], Dariusz Bartosik[1*]

[1]Department of Bacterial Genetics, Institute of Microbiology, Faculty of Biology, University of Warsaw, Miecznikowa 1, 02-096 Warsaw, Poland; aniaklicka@poczta.fm (AM), jczarnecki@biol.uw.edu.pl (JC), e_furmanczyk@poczta.fm (EF), lasek@biol.uw.edu.pl (RL), bajja@biol.uw.edu.pl (JB), bartosik@biol.uw.edu.pl (DB)

[2]Department of Environmental Microbiology and Biotechnology, Institute of Microbiology, Faculty of Biology, University of Warsaw, Miecznikowa 1, 02-096 Warsaw, Poland; ldziewit@biol.uw.edu.pl (LDz), ldrewniak@biol.uw.edu.pl (LDr), tkrucon@biol.uw.edu.pl (TK), kpiatkowska@biol.uw.edu.pl (KP)

[3]Department of Metabolic Regulation, Institute of Biochemistry, Faculty of Biology, University of Warsaw, Miecznikowa 1, 02-096 Warsaw, Poland; garstka@biol.uw.edu.pl (MG)

[4]Department of Plant Anatomy and Cytology, Institute of Experimental Plant Biology and Biotechnology, Faculty of Biology, University of Warsaw, Miecznikowa 1, 02-096 Warsaw, Poland; kat.gieczewska@biol.uw.edu.pl (KG)

[5]Bacterial Genome Plasticity, Department of Genomes and Genetics, Institut Pasteur, Paris, France

[6]Department of Plant Protection from Pests, Research Institute of Horticulture, Skierniewice, Poland

***Corresponding author:** bartosik@biol.uw.edu.pl

Department of Bacterial Genetics, Institute of Microbiology, Faculty of Biology, University of Warsaw, Miecznikowa 1, 02-096 Warsaw, Poland

**Table S1.** Absorbance and ESI$^{+*}$ mass spectrometry data for carotenoids produced by *P. marcusii* OS22.

| Retention time [min] | Estimated absorbance maxima [nm] (results accuracy ± 1 nm) | Estimated mass/charge ratio [m/z] (results accuracy 0.001 Da) | Molecular formula and compound name | Theoretical [m/z] ratio |
|---|---|---|---|---|
| 41-46 | 475 | 745.4680<br>767.4556 | C46H64O8H$^+$<br>C46H64O8Na<br>**Adonixanlhin-β-D-gIucoside** | 745.4680<br>767.4499 |
| 52-56 | 488 | 597.3955<br>619.3705 | $C_{40}H_{52}O_4H^+$<br>$C_{40}H_{52}O_4Na$<br>**Astaxanthin** | 597.3943<br>619.3763 |
| 74-76 | 470 | 583.4171<br>605.3943 | $C_{40}H_{54}O_3H^+$<br>$C_{40}H_{54}O_3Na$<br>**Adonixanthin** | 583.4151<br>605.3971 |
| 87 | 480 | 581.3990<br>603.3816 | $C_{40}H_{52}O_3H^+$<br>$C_{40}H_{52}O_3Na$<br>**Adonirubin** | 581.3995<br>603.3814 |
| 115, 204 | 464 | 567.4224<br>589.3967 | $C_{40}H_{54}O_2H^+$<br>$C_{40}H_{54}O_2Na$<br>**Hydroxyechinenone** | 567.4202<br>589.4022 |
| 129, 139-144 | 467-470 | 565.4009<br>587.3895 | $C_{40}H_{52}O_2H^+$<br>$C_{40}H_{52}O_2Na$<br>**Canthaxanthin** | 565.4045<br>587.3865 |
| 225-227 | 460 | 551.4238<br>573.4035 | $C_{40}H_{54}OH^+$<br>$C_{40}H_{54}ONa$<br>**Echinenone** | 551.4253<br>573.4072 |
| 240-247 | 447.475 | 553.4407 | $C_{40}H_{56}OH^+$<br>**Cryptoxanthin** | 553.4409 |
| 252 | 451.478 | 536.446 | $C_{40}H_{56}$<br>**β-carotene** | 536.4382 |

Carotenoids were identified based on characteristic absorption maxima and molecular masses.
* Electro Spray Ionization with positive mode

**Table S2.** Predicted genes of plasmid pCRT01.

| ORF no. | Coding region (gene orientation) | Protein size | Function | Best BLASTP hits | | |
|---|---|---|---|---|---|---|
| | | | | % of identity | Organism | GenBank accession no. |
| 1 | 67-606 (→) | 179 | Resolvase | 100 | *Paracoccus aminophilus* JCM 7686 (plasmid pAMI2) | YP_003208111 |
| 2 | 1126-2001 (→) | 291 | Replication protein RepA | 100 | *P. aminophilus* JCM 7686 (plasmid pAMI2) | YP_001965061 |
| 3 | 2230-2889 (→) | 219 | Partitioning protein ParA | 100 | *P. aminophilus* JCM 7686 (plasmid pAMI2) | YP_001965062 |
| 4 | 2886-3224 (→) | 112 | Partitioning protein ParB | 100 | *P. aminophilus* JCM 7686 (plasmid pAMI2) | YP_001965063 |
| 5 | 3308-3685 (→) | 125 | Addiction system toxin (Tad) | 100 | *P. aminophilus* JCM 7686 (plasmid pAMI2) | YP_001965064 |
| 6 | 3666-4001 (→) | 111 | Addiction system antitoxin (Ata) | 100 | *P. aminophilus* JCM 7686 (plasmid pAMI2) | YP_001965065 |
| 7 | 4088-4657 (→) | 189 | Hypothetical protein | 98 | *P. aminophilus* JCM 7686 (plasmid pAMI2) | YP_001965066 |
| 8 | 4695-5573 (←) | 292 | Conjugation protein TraG | 99 | *P. aminophilus* JCM 7686 (plasmid pAMI2) | YP_003208112 |
| 9 | 5835-6083 (←) | 82 | Hypothetical protein | 100 | *P. aminophilus* JCM 7686 (plasmid pAMI2) | YP_003208113 |
| 10 | 6158-6487 (←) | 109 | Conjugation protein MobC | 100 | *P. aminophilus* JCM 7686 (plasmid pAMI2) | YP_003208114 |
| 11 | 6881-10474 (→) | 1197 | Conjugation protein TraA | 99 | *P. aminophilus* JCM 7686 (plasmid pAMI2) | YP_003208115 |
| 12 | 10501-11070 (→) | 189 | Hypothetical protein | 100 | *P. aminophilus* JCM 7686 (plasmid pAMI2) | YP_003208116 |
| 13 | 11382-12469 (←) | 362 | Transposase IS*Pam3* | 100 | *P. aminophilus* JCM 7686 (plasmid pAMI2) | YP_003208117 |
| 14 | 13541-15829 (←) | 762 | *N,N*-dimethylformamidase, large subunit (DmfA2) | 99 | *P. aminophilus* JCM 7686 (plasmid pAMI2) | YP_003208118 |

| | | | | | | |
|---|---|---|---|---|---|---|
| 15 | 15825-16250 (←) | 141 | *N,N*-dimethylformamidase, small subunit (DmfA1) | 100 | *P. aminophilus* JCM 7686 (plasmid pAMI2) | YP_003208119 |
| 16 | 16282-17316 (←) | 344 | Transcription regulator DmfR | 100 | *P. aminophilus* JCM 7686 (plasmid pAMI2) | YP_003208120 |
| 17 | 17766-18537 (→) | 257 | Transposase IS*Pam4* | 100 | *P. aminophilus* JCM 7686 (plasmid pAMI2) | YP_003208121 |
| 18 | 18708-19079 (←) | 123 | Conjugation protein TraJ | 100 | Multispecies [Bacteria] | WP_008832110 |
| 19 | 19409-20014 (←) | 201 | Transposase IS*903* | 99 | *Escherichia coli* (plasmid pIS2) | YP_001687821 |
| 20 | 20151-20966 (→) | 271 | Kanamycin resistance gene | 100 | Multispecies [Bacteria] | WP_000018329 |
| 21 | 21667-22395 (→) | 242 | β-carotene oxygenase (CrtW) | 99 | *Paracoccus* sp. 228 | KIX17004 |
| 22 | 22392-22880 (→) | 162 | β-carotene hydroxylase (CrtZ) | 100 | *Paracoccus* sp. 228 | KIX17005 |
| 23 | 22877-24037 (→) | 386 | lycopene cyclase (CrtY) | 99 | *Paracoccus haeundaensis* | TNH40827 |
| 24 | 24034-25539 (→) | 501 | phytoene desaturase (CrtI) | 100 | *Paracoccus* sp. 228 | KIX17007 |
| 25 | 25536-26450 (→) | 304 | 15-*cis*-phytoene synthase (CrtB) | 100 | *Paracoccus* sp. 228 | KIX17008 |
| 26 | 26447-27328 (←) | 293 | geranylgeranyl diphosphate synthase (CrtE) | 100 | *Paracoccus* sp. 228 | WP_052715286 |
| 27 | 28936-29271 (→) | 111 | Hypothetical protein | 100 | *Salmonella enterica* G8430 (plasmid pU302S) | YP_194804 |
| 28 | 29704-30475 (→) | 257 | Transposase IS*Pam4* | 100 | *P. aminophilus* JCM 7686 (plasmid pAMI2) | YP_003208121 |

**Table S3.** Quantitative analysis of carotenoids identified in extracts of *Paracoccus* spp. cells.

| Carotenoid | Carotenoids concentration [mol%] in cell extracts of: | | |
|---|---|---|---|
| | *P. marcusii* OS22 | *P. aminophilus* CRT1 | *P. kondratievae* CRT2 |
| β-carotene | 3.80 ± 1.72 | 72.18 ± 13.73 | 12.39 ± 4.37 |
| Echinenone | 22.79 ± 0.73 | 14.78 ± 12.27 | 18.87 ± 14.05 |
| Cryptoxanthin | 0.43 ± 0.16 | 5.45 ± 1.02 | 0.88 ± 0.24 |
| Hydroxyechinenone | 13.68 ± 0.08 | 3.10 ± 2.78 | 13.96 ± 9.24 |
| Canthaxanthin | 15.42 ± 2.70 | 1.65 ± 1.80 | 5.02 ± 2.79 |
| Adonixanthin | 22.19 ± 0.94 | 1.18 ± 1.18 | 38.45 ± 17.24 |
| Adonirubin | 11.33 ± 0.18 | 1.66 ± 1.30 | 4.21 ± 0.80 |
| Astaxanthin | 6.59 ± 0.42 | 0.00 ± 0.00 | 6.22 ± 3.59 |
| Adonixanthin-β-D-gIucoside | 3.78 ± 0.45 | 0.00 ± 0.00 | 0.00 ± 0.00 |

Carotenoid concentrations were calculated from the calibration curve prepared for β-carotene. Absorption coefficients ε = 139 / (mM cm) at λ = 453 nm was used. The data show mean values ± SD for 3 to 4 experiments.

**Table S4.** The content of anions and elements in flue gas desulfurization (FGD) wastewater.

| **Anions** (concentrtion [mg $L^{-1}$]) | **Element** (concentration [μg $L^{-1}$]) |
|---|---|
| $NH_4^+$ (46.90); Br- (16.20); $Cl^-$ (840); $NO^{3-}$(130); $SO_4^{2-}$ (7090); $NO^{2-}$ (<0.20); $F^-$ (78.50); $PO_4^{3-}$ (<2.50) | Quantative: Al (477.52); As (7.31); Cr (1.56); Cu (15.15); Fe (720.62); Ni (383.46); Se (703.15); V (8.02); Zn (369.40);<br><br>Semi-quantative: B (419.97); Ba (59.96); Cd (44.28); Co (63.24); Mo (14.10); Rb (29.60); Sr (924.58); Tl (4.05); U (13.14) |

**Table S5.** Standard deviation (%) of growth parameters of *Paracoccus* spp. producing carotenoids.

| Growth medium | *Paracoccus aminophilus* CRT1 | | | | *Paracoccus kondratievae* CRT2 | | | |
|---|---|---|---|---|---|---|---|---|
| | **Time of cultivation [h]** | | | | **Time of cultivation [h]** | | | |
| | **0** | **24** | **48** | **72** | **0** | **24** | **48** | **72** |
| | **pH** | | | | | | | |
| **M9/methanol** | 0.50 | 0.16 | 0.13 | 0.21 | 0.50 | 0.30 | 0.13 | 0.04 |
| **M9/molasses** | 0.50 | 0.10 | 3.79 | 4.91 | 0.50 | 0.86 | 4.50 | 8.03 |
| **M9** | 5.00 | 5.00 | 5.00 | 5.00 | 0.50 | 0.50 | 0.50 | 0.50 |
| **FGD/methanol** | 3.65 | 4.07 | 1.83 | 0.15 | 2.80 | 1.22 | 0.99 | 0.10 |
| **FGD/molasses** | 0.50 | 1.76 | 5.08 | 4.05 | 0.50 | 0.30 | 1.13 | 0.27 |
| **FGD** | 5.00 | 5.00 | 5.00 | 5.00 | 0.50 | 0.50 | 0.50 | 0.50 |
| **LB** | 5.00 | 5.00 | 5.00 | 5.00 | 0.50 | 0.50 | 0.50 | 0.50 |
| | **CFU $mL^{-1}$** | | | | | | | |
| **M9/methanol** | 6.52 | 1.87 | 0.97 | 3.37 | 6.16 | 1.54 | 2.88 | 3.19 |
| **M9/molasses** | 0.99 | 5.50 | 2.16 | 2.76 | 0.25 | 0.83 | 0.82 | 0.90 |
| **M9** | 6.06 | 0.00 | 0.00 | 0.00 | 7.33 | 10.00 | 0.00 | 0.00 |
| **FGD/methanol** | 0.08 | 3.15 | 7.39 | 2.94 | 2.94 | 4.67 | 2.05 | 3.41 |
| **FGD/molasses** | 0.00 | 1.20 | 6.12 | 9.78 | 4.64 | 1.73 | 5.09 | 7.30 |
| **FGD** | 1.11 | 4.79 | 4.94 | 8.77 | 5.52 | 5.28 | 5.21 | 0.00 |
| **LB** | 43.69 | 3.74 | 1.27 | 0.00 | 3.87 | 0.00 | 0.00 | 0.00 |
| | **sCOD [mg $L^{-1}$]** | | | | | | | |
| **M9/methanol** | 30.41 | 12.84 | 8.20 | 17.41 | 1.69 | 3.39 | 3.70 | 5.50 |
| **M9/molasses** | 5.96 | 11.27 | 3.61 | 9.62 | 11.35 | 9.26 | 3.90 | 15.59 |
| **M9** | 10.00 | 10.00 | 10.00 | 10.00 | 10.00 | 10.00 | 10.00 | 10.00 |
| **FGD/methanol** | 3.51 | 5.95 | 4.09 | 11.83 | 2.58 | 1.57 | 4.97 | 6.27 |
| **FGD/molasses** | 6.53 | 11.50 | 3.19 | 10.48 | 12.51 | 10.00 | 3.45 | 17.03 |
| **FGD** | 10.00 | 10.00 | 10.00 | 10.00 | 10.00 | 10.00 | 10.00 | 10.00 |
| **LB** | 10.00 | 10.00 | 10.00 | 10.00 | 10.00 | 10.00 | 10.00 | 10.00 |

**Table S6.** Bacterial strains, plasmids and oligonucleotides used in this study.

| **Bacterial strains** | **Relevant characteristics/Genotype** | **Source** |
|---|---|---|
| *Paracoccus marcusii* OS22 | Carotenoids producing environmental strain | [1] |
| *Paracoccus aminophilus* JCM 7686R | Rif$^r$ derivative of a wild type strain JCM 7686; contains native plasmid pAMI2 | [2] |
| *Paracoccus kondratievae* NCIBM 131773R | Rif$^r$ derivative of a wild type strain NCIBM 131773 | [3] |
| *Paracoccus versutus* UW225 | Rif$^r$ derivative of a wild type strain UW1, deprived of native plasmid pTAV1 | [4] |
| *E. coli* DH5α | F$^-$ φ80*lac*ZΔM15 Δ(*lac*ZYA-*arg*F)U169 *rec*A1 *end*A1 *hsd*R17(r$_K^-$, m$_K^+$) *pho*A *sup*E44 λ$^-$ *thi*-1 *gyr*A96 *rel*A1 | [5] |
| *E. coli* DH5αpir | *endA*1 *hsdR*17 *glnV*44 (= *supE*44) *thi*-1 *recA*1 *gyrA*96 *relA*1 φ80d*lac*Δ(l*acZ*)M15 Δ(*lacZYA-argF*)U169 *zdg*-232::Tn10 *uidA*::*pir*+ | [6] |
| *E. coli* S-17.1 | Tp$^r$ Sm$^r$ *recA*, *thi*, *pro*, *hsdR*-M+RP4: 2-Tc:Mu: Km Tn*7* λpir | [7] |
| *P. aminophilus* CRT1 | JCM 7686R containing pCRT01 | this study |
| *P. kondratievae* CRT2 | NCIBM 131773R containing pCRT01 | this study |
| **Plasmids** | **Description** | **Source** |
| pABW1 | Cloning vector; 4,5 kpz; Km$^r$, *ori* pMB1, *oriT* RK2; *lac*Zα; MCS | |
| pABW1-crtW | pABW1 carrying OS22 *crtW* gene (amplified by PCR with primers CRTWL and CRTWR) inserted in XbaI site | this study |
| pABW1-ctr | pABW1 carrying *crt* gene cluser of *P. marcusii* OS22 | this study |
| pCRT01 | Co-integrate plasmid containing pABW1-crt and pAMI2 | this study |
| pCM132 | Promoter probe vector; 11,3 kpz; Km$^r$, *ori* pMB1, *ori* RK2, *oriT* RK2, *lacZ* reporter gene | [8] |
| pCM-PW | pCM132 carrying upstream region of OS22 *crtW* gene (amplified with primers PCRTWE and PCRTWB) inserted in EcoRI and BglII sites | this study |
| pCM-PZ | pCM132 carrying upstream region of OS22 *crtZ* gene (amplified with primers PCRTZLE and PCRTZRB inserted in EcoRI and BglII sites | this study |
| pCM-PY | pCM132 carrying upstream region of OS22 *crtY* gene (amplified with primers PCRTYLE and PCRTYRB inserted in EcoRI and BglII sites | this study |
| pCM-PI | pCM132 carrying upstream region of OS22 *crtI* gene (amplified with primers PCRTILE and PCRTIRB inserted in EcoRI and BglII sites | this study |
| pCM-PB | pCM132 carrying upstream region of OS22 *crtB* gene (amplified with primers PCRTBLE and PCRTBRB inserted in EcoRI and BglII sites | this study |
| pCM-PE | pCM132 carrying upstream region of OS22 *crtE* gene (amplified with primers PCRTELE and PCRTERB inserted in EcoRI and BglII sites | this study |
| pUT-Km | Tn5-based delivery plasmid with Km$^r$, Ap$^r$; *oriγ* R6K, *oriT* RK2, *tnp** gene of Tn*5*-IS*50*R | [9] |

| | | |
|---|---|---|
| pDIY-Km | Source of Km$^r$ cassette; *ori* pMB1 | [10] |
| pRK2013 | Km$^r$; helper plasmid carrying genes for conjugal transfer of RK2 | [11] |
| pUToriγKm | pUT-Km carrying Km$^r$ cassette (derived from pDIY-KM) and *ori*γ of R6K inserted within transposition cassette | this study |
| **Oligonucleotides** | **DNA Sequence (5′→3′)** | **Features** |
| CRTWF | TCTAGAGGCCAATGGTCGCAAGCAAC | XbaI |
| CRTWR | TCTAGAGTGGCGACGACGATCAGGAA | XbaI |
| FPUT | TGGTTGTAACACTGGCAGAG | |
| RPUT | AATCGCGGCCTCGAGCAAGA | |
| PCRTWE | GGCGAATTCAGGAAGGTGGCGACCGCAAT | EcoRI |
| PCRTWB | GGCAGATCTGGCATGTGCGCTCATCTTGT | BglII |
| PCRTZLE | GGCGAATTCGGATCGTTGGATGTACGTGG | EcoRI |
| PCRTZRB | TGGATCCGGAATAGGCCGTCAACTCCA | BamHI |
| PCRTYLE | GGCGAATTCGTGATCGCCACGGTGCTGTT | EcoRI |
| PCRTYRB | TGGATCCGGCAAGGTCCAGCAACAGCA | BamHI |
| PCRTILE | GGCGAATTCATTCCGATGGCGGCGATCTG | EcoRI |
| PCRTIRB | TGGATCCAAGCCTGCGCCGATCACGAT | BamHI |
| PCRTELE | GGCGAATTCCGGATGCCTGGTGATCGGA | EcoRI |
| PCRTERB | TGGATCCGGCGATCTCCTCAAGTCTGG | BamHI |

References to Table S6

[1] Drewniak L, Styczek A, Majder-Lopatka M, Sklodowska A. Bacteria hypertolerant to arsenic in the rocks of an ancient gold mine, and their potential role in dissemination of arsenic pollution. Environ Pollut. 2008;156(3):1069-74.

[2] Dziewit L, Jazurek M, Drewniak L, Baj J, Bartosik D. The SXT conjugative element and linear prophage N15 encode toxin-antitoxin-stabilizing systems homologous to the tad-ata module of the *Paracoccus aminophilus* plasmid pAMI2. J Bacteriol. 2007;189(5):1983-97.

[3] Dziewit L, Baj J, Szuplewska M, Maj A, Tabin M, Czyzkowska A, Skrzypczyk G, Adamczuk M, Sitarek T, Stawinski P et al. Insights into the transposable mobilome of *Paracoccus* spp. (*Alphaproteobacteria*). PLoS One. 2012;7(2):e32277.

[4] Bartosik D, Baj J, Plasota M, Piechucka E, Wlodarczyk M. Analysis of *Thiobacillus versutus* pTAV1 plasmid functions. Acta Microbiol Polon. 1993;39:5-15.

[5] Gibson TJ. Studies on Epstein-Barr genome. PhD thesis, University of Cambridge. 1984.

[6] Platt R, Drescher C, Park SK, Phillips GJ. Genetic system for reversible integration of DNA constructs and *lacZ* gene fusions into the *Escherichia coli* chromosome. Plasmid. 2000;43(1):12-23.

[7] Priefer UB, Simon R, Puhler A. Extension of the host range of Escherichia coli vectors by incorporation of RSF1010 replication and mobilization functions. J Bacteriol. 1985;163(1):324-30.

[8] Marx CJ, Lidstrom ME. Development of improved versatile broad-host-range vectors for use in methylotrophs and other Gram-negative bacteria. Microbiology. 2001;147(Pt 8):2065-75.

[9] de Lorenzo V, Herrero M, Jakubzik U, Timmis KN. Mini-Tn5 transposon derivatives for insertion mutagenesis, promoter probing, and chromosomal insertion of cloned DNA in gram-negative eubacteria. J Bacteriol. 1990;172(11):6568-72.

[10] Dziewit L, Adamczuk M, Szuplewska M, Bartosik D. DIY series of genetic cassettes useful in construction of versatile vectors specific for *Alphaproteobacteria*. J Microbiol Methods. 2011;86(2):166-74.

[11] Ditta G, Stanfield S, Corbin D, et al. Broad host range DNA cloning system for gram-negative bacteria: construction of a gene bank of *Rhizobium meliloti*. Proc Natl Acad Sci USA. 1980;77:7347-51.
